# Supplementary material for: Substantial hysteresis in emergent temperature sensitivity of global wetland CH4 emissions
Source: Nat Commun. 2021 Apr 15;12:2266. doi: 10.1038/s41467-021-22452-1 (PMC8050324; doi:10.1038/s41467-021-22452-1)
Supplement: Supplementary file 1 — Supplementary Information [file 41467_2021_22452_MOESM1_ESM.pdf]

Supplementary Information for

Substantial hysteresis in emergent temperature sensitivity of global wetland CH_4 emissions

Kuang-Yu Chang et al.

Correspondence to: Kuang-Yu Chang (ckychang@lbl.gov)

Supplemental Table 1. Characteristics of wetland and rice paddy sites currently included in the FLUXNET-CH_4 database. LAT and LON stand for latitude and longitude, respectively.

Site ID	Site Name	Country	LAT	LON	Biome	Ecosystem Type	Site PI	DOI/ Dataset
CA-SCB	Scotty Creek Bog	Canada	61.31	-121.30	Taiga	Bog	Sonnentag & Helbig	doi.org/10.17190/AMF/1498754
CA-SCC	Scotty Creek Peat plateau	Canada	61.31	-121.30	Taiga	Peat plateau; forest-bog	Sonnentag & Helbig	doi.org/10.17190/AMF/1480303
DE-SfN	Schechenfilz Nord	Germany	47.81	11.33	Temperate	Bog	Schmid	European Fluxes Database Cluster
DE-Zrk	Zarnekow	Germany	53.88	12.89	Temperate	Fen	Sachs	European Fluxes Database Cluster
FI-Lom	Lompolojankka	Finland	68.00	24.21	Taiga	Fen	Lohila & Aurela	European Fluxes Database Cluster
FI-Si2	Siikaneva II	Finland	61.84	24.17	Taiga	Bog	Vesala & Mammarella	European Fluxes Database Cluster
FI-Sii,	Siikaneva I	Finland	61.83	24.19	Taiga	Fen	Vesala & Mammarella	European Fluxes Database Cluster
IT-Cas	Castellaro	Italy	45.07	8.72	Temperate	Rice	Cescatti	European Fluxes Database Cluster
JP-BBY	Bibai Mire	Japan	43.32	141.81	Temperate	Bog	Ueyama	European Fluxes Database Cluster
JP-Mse	Mase paddy flux site	Japan	36.05	140.03	Temperate	Rice	Miyata	European Fluxes Database Cluster
KR-CRK	Cheorwon Rice paddy South	South Korea	38.20	127.25	Temperate	Rice	Ryu & Kang	European Fluxes Database Cluster
MY-MLM	Maludam	Malaysia	1.45	111.15	Tropical& Subtropical	Swamp	Tang	https://doi.org/10.5281/zenodo.1161966
NZ-Kop	Kopuatai	New Zealand	-37.39	175.55	Temperate	Bog	Campbell	DOI: 10.18140/FLX/1669652
RU-Ch2	Chersky reference	Russia	68.62	161.35	Taiga	Wet tundra	Goeckede	European Fluxes Database Cluster
RU-Che	Chersky	Russia	68.61	161.34	Taiga	Wet tundra	Goeckede	European Fluxes Database Cluster
RU-SAM	Samoylov	Russia	72.37	126.50	Tundra	Wet tundra	Sachs	European Fluxes Database Cluster
RU-Vrk	Seida/Vorkuta	Russia	67.06	62.94	Tundra	Wet tundra	Friborg	European Fluxes Database Cluster
SE-Deg	Degero	Sweden	64.18	19.56	Taiga	Fen	Nilsson & Peichl	European Fluxes Database Cluster
SE-St1	Stordalen grassland (Mire)	Sweden	68.35	19.05	Tundra	Fen	Friborg	European Fluxes Database Cluster
SE-Sto	Stordalen Palsa Bog	Sweden	68.36	19.05	Tundra	Bog	Friborg	European Fluxes Database Cluster
US-Atq	Atqasuk	USA	70.47	-157.41	Tundra	Wet tundra	Zona	doi:10.17190/AMF/1246029
US-Beo	Barrow	USA	71.28	-156.61	Tundra	Wet tundra	Donatella Zona	DOI:10.18140/FLX/1669664
US-Bes	Barrow	USA	71.28	-156.60	Tundra	Wet tundra	Zona	DOI:10.18140/FLX/1669665
US-Bgl	Bog Lake peatland	USA	47.53	-93.74	Temperate	Fen	Verma	AmeriFlux

US-Bzb	Thermokarst collapse scar bog	USA	64.7	-148.32	Taiga	Bog	Euskirchen	https://doi.org/10.18140/FLX/1669668
US-Bzf	Rich Fen	USA	64.70	-148.313	Taiga	Fen	Euskirchen	doi.org/10.17190/AMF/1756433
US-HRA	Humnoke Farm Rice Field AWD	USA	34.59	-91.75	Temperate	Rice	Runkle	doi.org/10.17190/AMF/1543376
US-HRC	Humnoke Farm Rice Field conventional	USA	34.59	-91.75	Temperate	Rice	Reba	doi.org/10.17190/AMF/1543375
US-Ics	Wet sedge tundra	USA	68.61	-149.31	Tundra	Wet tundra	Euskirchen	doi: 10.17190/AMF/1246130
US-Ivo	Ivotuk	USA	68.49	-155.75	Tundra	Wet tundra	Zona	doi:10.17190/AMF/1246067
US-LA1	Pointe-aux-Chenes Brackish Marsh	USA	29.50	-90.45	Subtropical	Salt Marsh	Krauss & Holm	doi.org/10.17190/AMF/1543386
US-LA2	Salvador WMA Freshwater Marsh	USA	29.86	-90.29	Subtropical	Marsh	Krauss & Holm	doi.org/10.17190/AMF/1543387
US-Los	Lost Creek	USA	46.08	-89.98	Temperate	Fen	Desai	doi: 10.17190/AMF/1246071
US-MRM	Marsh Resource Meadowlands Mitigation Bank	USA	40.82	-74.04	Temperate	Salt Marsh	Schäfer	DOI:10.18140/FLX/1669684
US-Myb	Mayberry Wetland	USA	38.05	-121.77	Temperate	Marsh	Baldocchi	doi: 10.17190/AMF/1246139
US-NC4	NC Alligator River	USA	35.79	-75.90	Temperate	Swamp	Noormets	doi:10.17190/AMF/1480314
US-NGB	NGEE Barrow	USA	71.28	-156.61	Tundra	Wet tundra	Torn	doi: 10.17190/AMF/1436326
US-NGC	NGEE Arctic Council	USA	64.86	-163.70	Taiga	Wet tundra	Torn	doi.org/10.17190/AMF/1634883
US-ORv	Olentangy River Wetland Research Park	USA	40.02	-83.02	Temperate	Marsh	Bohrer	doi:10.17190/AMF/1246135
US-OWC	Old Woman Creek	USA	41.38	-82.51	Temperate	Marsh	Bohrer	doi: 10.17190/AMF/1246094
US-Sne	Sherman Island Restored Wetland	USA	38.04	-121.76	Temperate	Marsh	Baldocchi	doi: 10.17190/AMF/1418684
US-Srr	Suisun marsh - Rush Ranch	USA	38.20	-122.03	Temperate	Salt Marsh	Windham-Myers	doi:10.17190/AMF/1418685
US-StJ	St Jones Reserve	USA	39.09	-75.44	Temperate	Salt Marsh	Vargas	doi:10.17190/AMF/1480316
US-Tw1	Twitchell West Pond Wetland	USA	38.11	-121.65	Temperate	Marsh	Baldocchi	doi: 10.17190/AMF/1246147
US-Tw4	Twitchell East End	USA	38.10	-121.64	Temperate	Marsh	Baldocchi	doi: 10.17190/AMF/1246148

	Wetland							
US-Twt	Twitchell Rice	USA	38.11	-121.65	Temperate	Rice	Baldocchi	doi: 10.17190/AMF/1246151
US-Uaf	University of Alaska, Fairbanks	USA	64.87	-147.86	Taiga	Bog	Ueyama	doi:10.17190/AMF/1480322
US-WPT	Winous Point North Marsh	USA	41.46	-83.00	Temperate	Marsh	Chu	doi: 10.17190/AMF/1246155

Supplemental Table 2. Description of model configurations used in the six sets of regression models representing varying degrees of spatial and temporal variability between CH_4 emission and air temperature.

	Model group 1 *f(T, site,IAV,ISV)*	Model group 2 *f(T, site,IAV)*	Model group 3 *f(T,site)*	Model group 4 *f(T,type,ISV)*	Model group 5 *f(T,type)*	Model group 6 *f(T)*
Temperature	Yes	Yes	Yes	Yes	Yes	Yes
Inter-type variability	Yes	Yes	Yes	Yes	Yes	No
Inter-site variability	Yes	Yes	Yes	No	No	No
Inter-annual variability	Yes	Yes	No	No	No	No
Intra-seasonal variability	Yes	No	No	Yes	No	No

Emergent temperature responses may change during the course of the frost-free season within the same site-year (i.e., intra-seasonal variability), among frost-free seasons within the same ecosystem site (i.e., inter-annual variability), among sites within the same ecosystem type (i.e., inter-site variability), and among ecosystem types (i.e., inter-type variability).

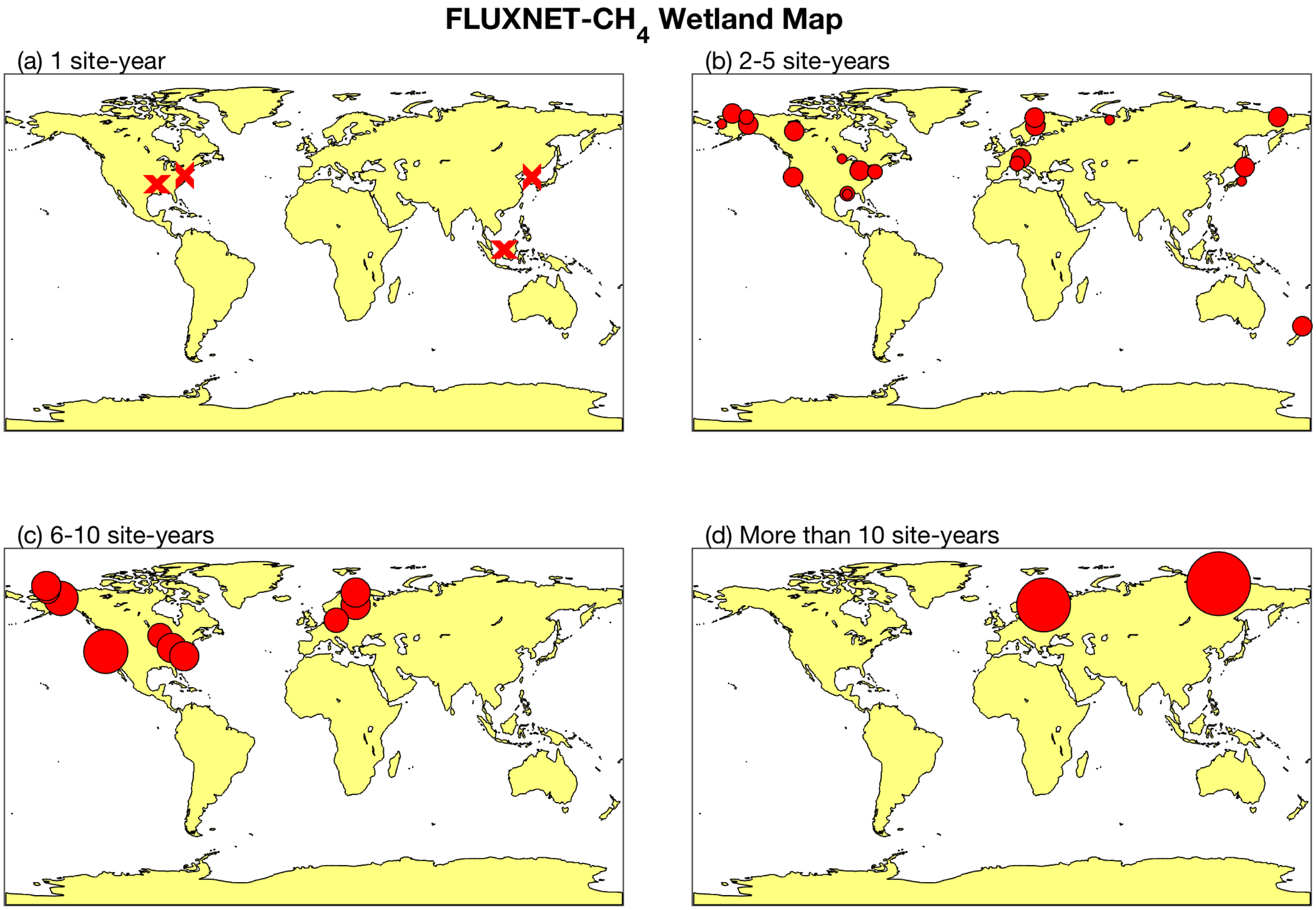

Supplemental Figure 1. **Geographical locations of wetland and rice paddy sites currently included in the FLUXNET-CH_4 database.** For sites that have measurement records for a single site-year (a), 2 - 5 site-years (b), 6 - 10 site-years (c), and more than 10 site-years (d). Circle sizes are proportional to the number of site-years at each site (1b to 1d).

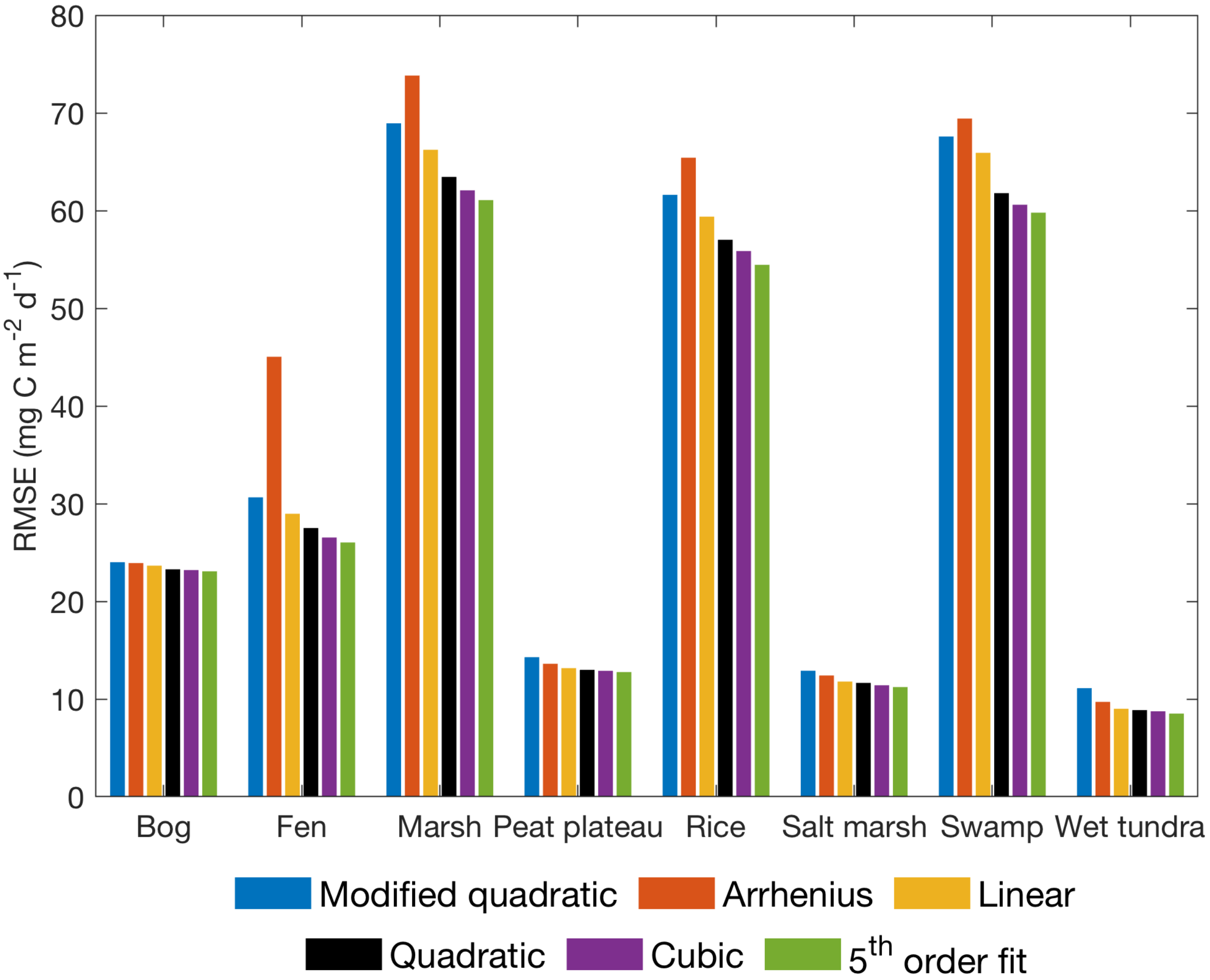

Supplemental Figure 2. **The Root mean squared errors (RMSE) for daily CH_4 emission predictions are comparable among temperature dependence models.** RMSE comparison among daily CH_4 emissions estimated by temperature dependence models based on different curve fitting schemes in each ecosystem type. The abbreviations used in each curve fitting class represent the quadratic equation used in this study (Modified quadratic), the Boltzmann-Arrhenius equation used in this study (Arrhenius), first order polynomial fit (Linear), second order polynomial fit (Quadratic), third order polynomial fit (Cubic), and fifth order polynomial fit (5th order fit).

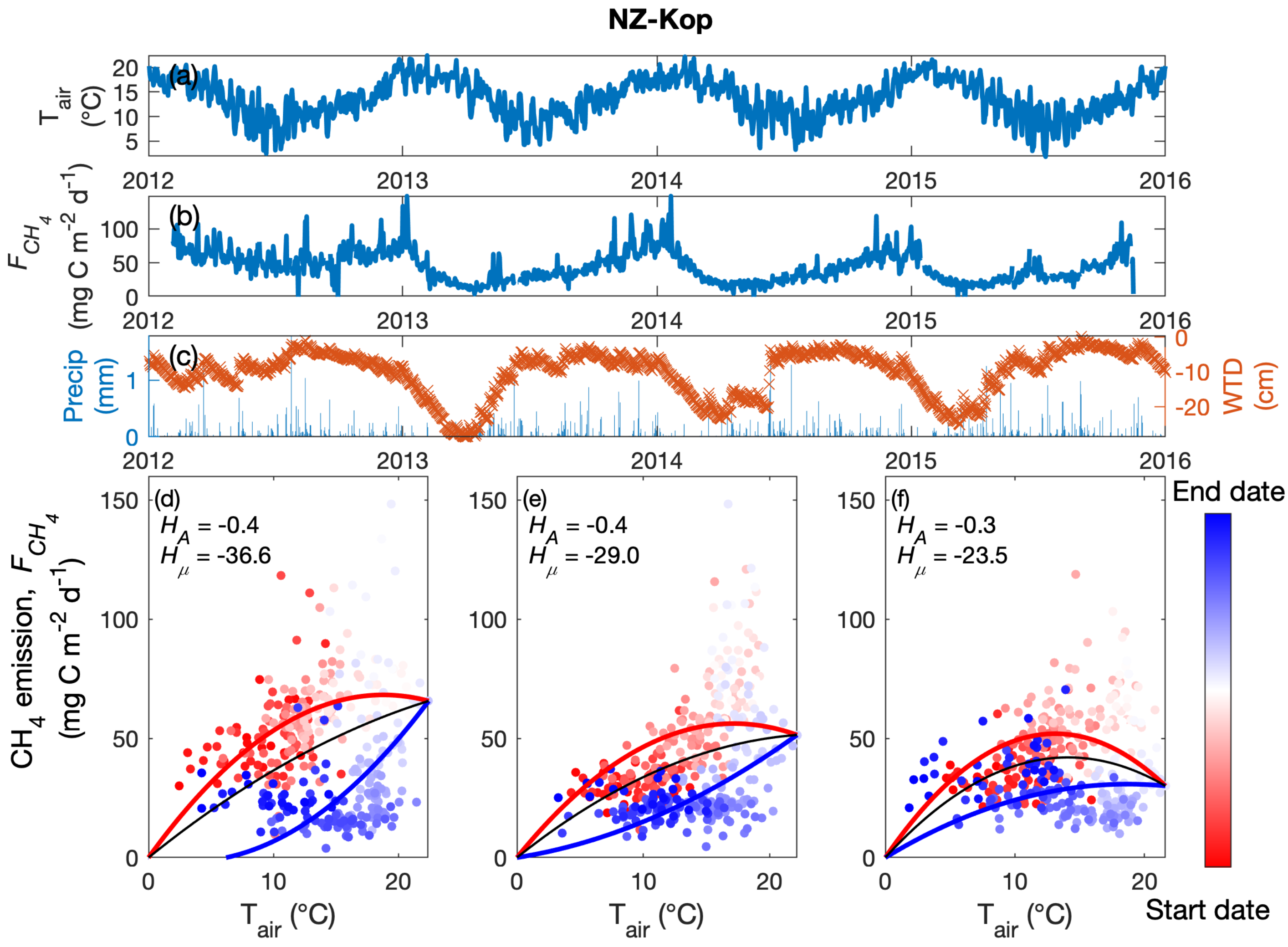

Supplemental Figure 3. **Negative seasonal F_{CH_4} hysteresis can occur when WTD drops below the critical zone of CH_4 production later in the frost-free season.** The quality-controlled daily air temperature (a) and CH_4 emissions (b), precipitation (c, left axis), and water table depth (c, right axis) measured at Kopuatai in New Zealand (NZ-Kop) from 2012 to 2015. CH_4 emission air temperature dependencies (lines) derived from daily measurements (dots) recorded at NZ-Kop for 2012–2013 (d), 2013–2014 (e), and 2014–2015 (f). The results inferred from earlier and later parts of the frost-free season, and full frost-free season are colored in red, blue, and black, respectively. Start and end dates represent the beginning and ending of the frost-free season, respectively. H_μ and H_A denote the mean seasonal CH_4 emission hysteresis and normalized area of seasonal CH_4 emission hysteresis calculated in each site-year, respectively. Note that site-years were defined by warming and cooling cycles (i.e., a site-year is composed by the warming branch in the previous calendar year and the

cooling branch in the current calendar year) to account for the opposite seasonal cycle in the Southern compared to the Northern Hemisphere.

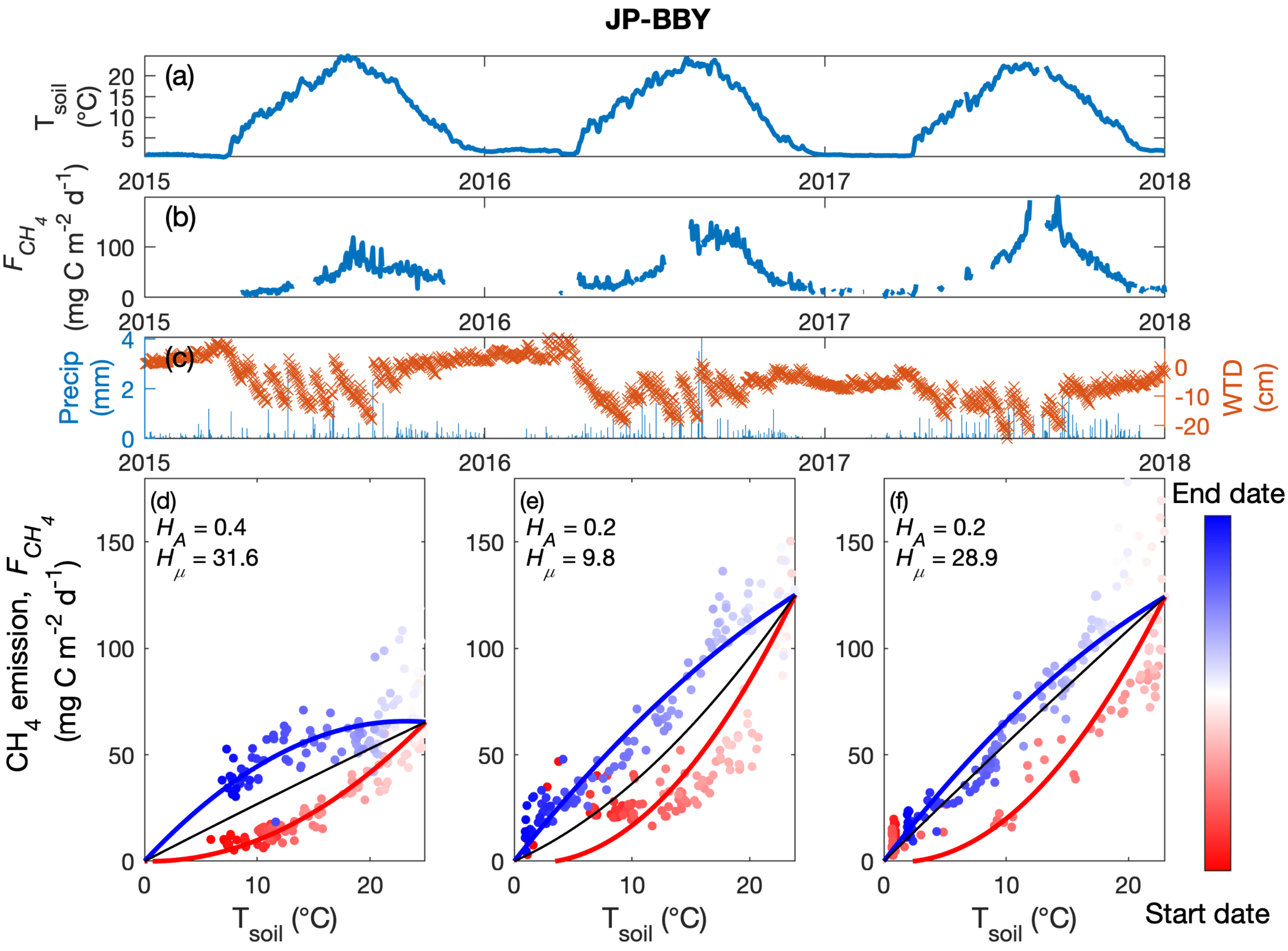

Supplemental Figure 4. **The hysteretic relationship between CH_4 emission and soil temperature is consistent with the patterns found using air temperature.** The same as Fig. 1 except using soil temperature. The quality-controlled daily soil temperature measured at the shallowest soil layer (a), CH_4 emissions (b), precipitation (c, left axis), and water table depth (c, right axis) measured at the Bibai Mire in Japan (JP-BBY) from 2015 to 2017. CH_4 emission soil temperature dependencies (lines) derived from daily measurements (dots) recorded at JP-BBY for 2015 (d), 2016 (e), and 2017 (f). The results inferred from earlier and later parts of the frost-free season, and full frost-free season are colored in red, blue, and black, respectively. Start and end dates represent the beginning and ending of the frost-free season, respectively. H_μ and H_A denote the mean seasonal CH_4 emission hysteresis and normalized area of seasonal CH_4 emission hysteresis calculated in each site-year, respectively.

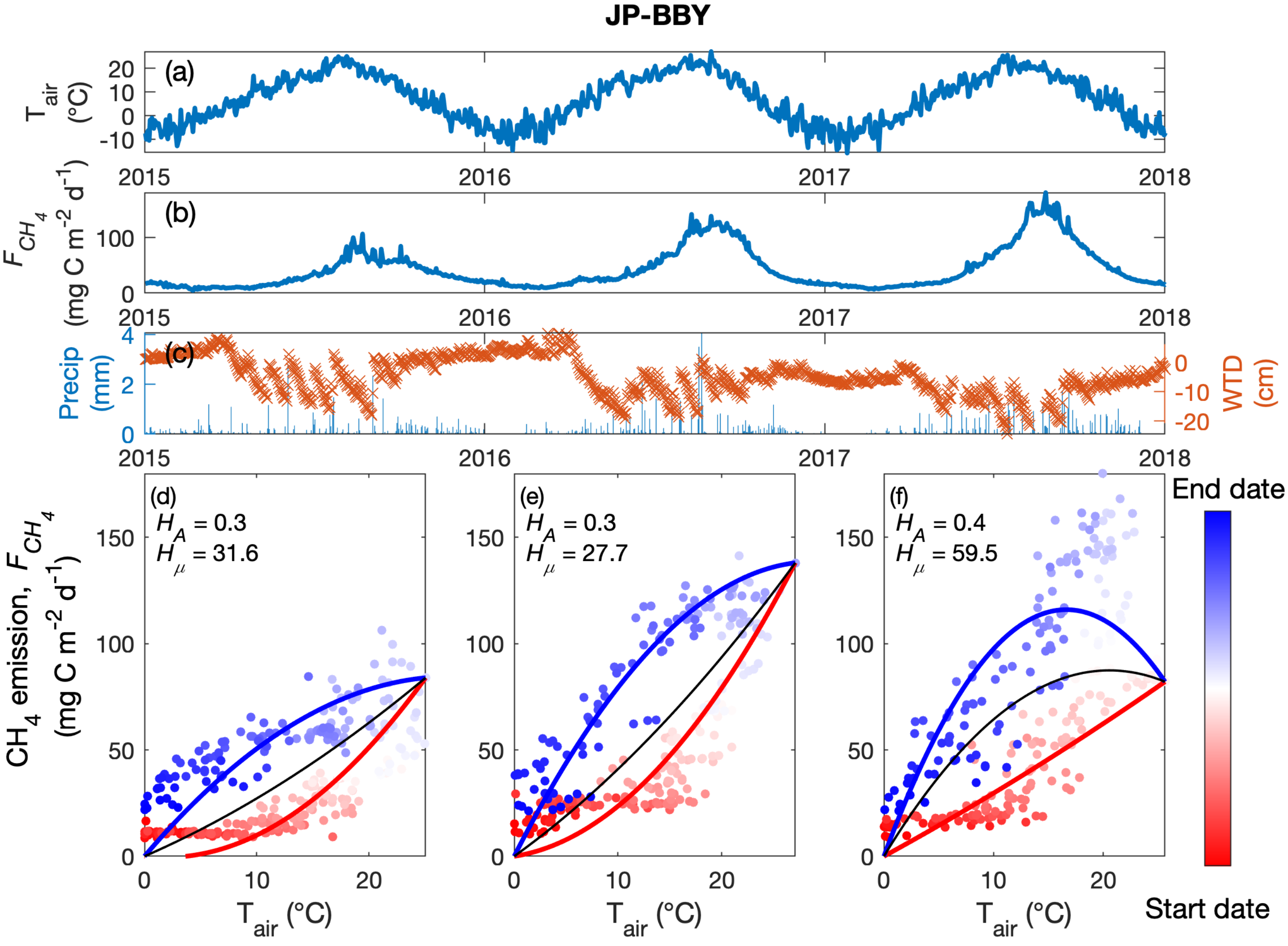

Supplemental Figure 5. **The hysteretic relationship between gap-filled CH_4 emission and air temperature is consistent with the patterns found using non-gap-filled data.** The same as Fig. 1 except using gap-filled data. The daily air temperature (a), CH_4 emissions (b) gap-filled by Artificial Neural Networks, and precipitation (c, left axis), and water table depth (c, right axis) measured at the Bibai Mire in Japan (JP-BBY) from 2015 to 2017. CH_4 emission air temperature dependencies (lines) derived from daily measurements (dots) recorded at JP-BBY for 2015 (d), 2016 (e), and 2017 (f). The results inferred from earlier and later parts of the frost-free season, and full frost-free season are colored in red, blue, and black, respectively. Start and end dates represent the beginning and ending of the frost-free season, respectively. H_μ and H_A denote the mean seasonal CH_4 emission hysteresis and normalized area of seasonal CH_4 emission hysteresis calculated in each site-year, respectively.

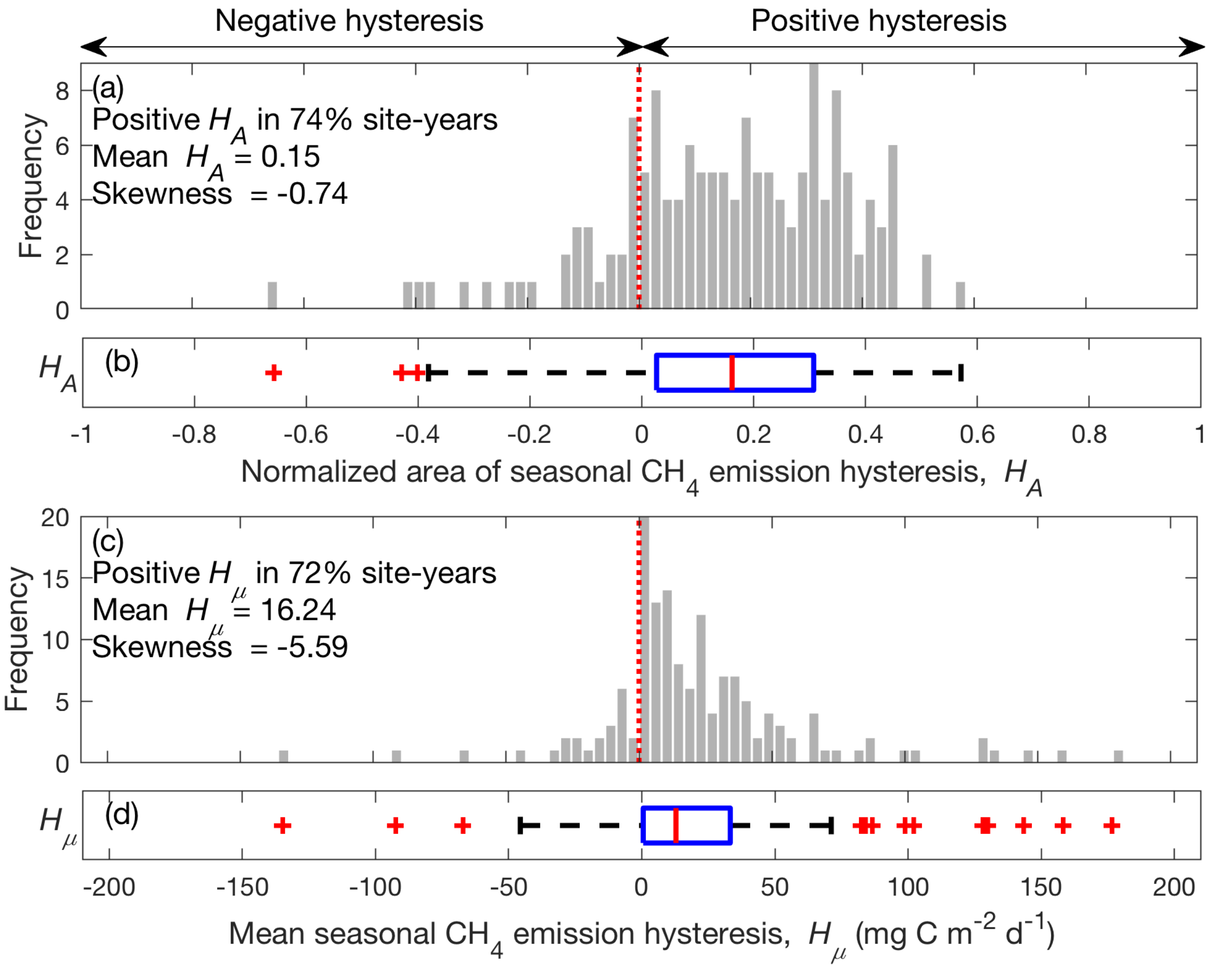

Supplemental Figure 6. **The distribution of seasonal CH_4 emission hysteresis inferred from monthly estimates is consistent with the patterns found using daily estimates.** The distribution of normalized area of seasonal CH_4 emission hysteresis (H_A; a, b) and mean seasonal CH_4 emission hysteresis (H_μ; c, d) to air temperature among site-years derived from the FLUXNET-CH_4 database. Positive seasonal CH_4 emission hysteresis indicates higher CH_4 emissions later in the frost-free season at the same temperature (e.g., Fig. 1d, e, f). Red dashed lines represent the y-axis in each data group (i.e., no hysteresis). The corresponding boxplot of site-year specific H_A (b) and H_μ (d) derived from the FLUXNET-CH_4 database. The red central mark, and the bottom and top edges of the blue box indicate the median, and the 25th and 75th percentiles, respectively. The black whiskers extend to the most extreme data points not considered outliers denoted in red plus symbol.

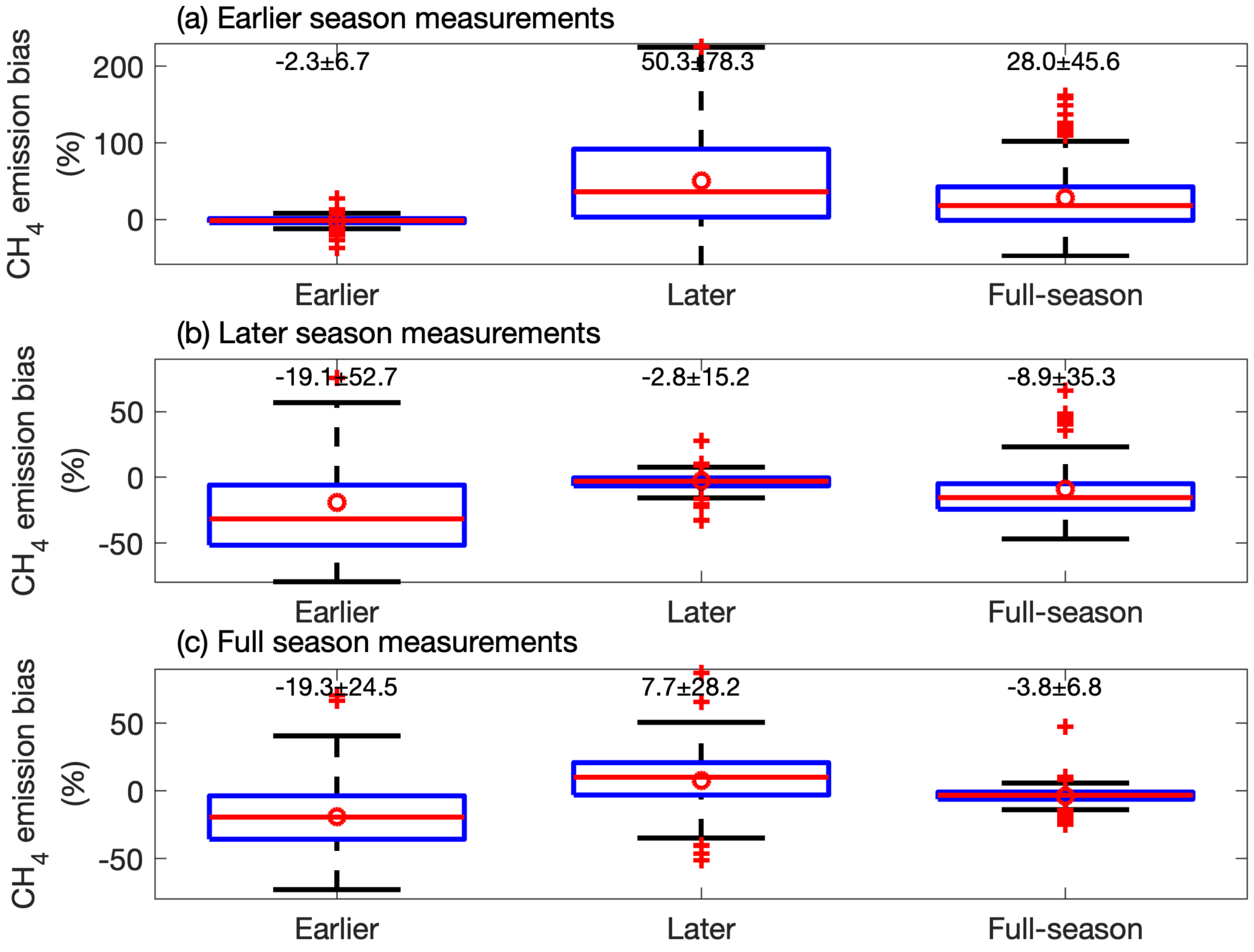

Supplemental Figure 7. **The accuracy of CH_4 emission predictions made by emergent air temperature dependence on CH_4 emission relies on the sampling period of the frost-free season.** CH_4 emission bias associated with using emergent air temperature dependence models inferred from earlier, later, and full-season measurements taken from a frost-free season during the earlier (a), later (b), and full-season periods (c) across global wetland and rice paddy sites. The red central mark and open circle, and the bottom and top edges of the blue box indicate the median and mean, and the 25th and 75th percentiles, respectively. The black whiskers extend to the most extreme data points not considered outliers denoted in red plus symbol(s). Numbers above each boxplot indicate the mean ± standard deviation.

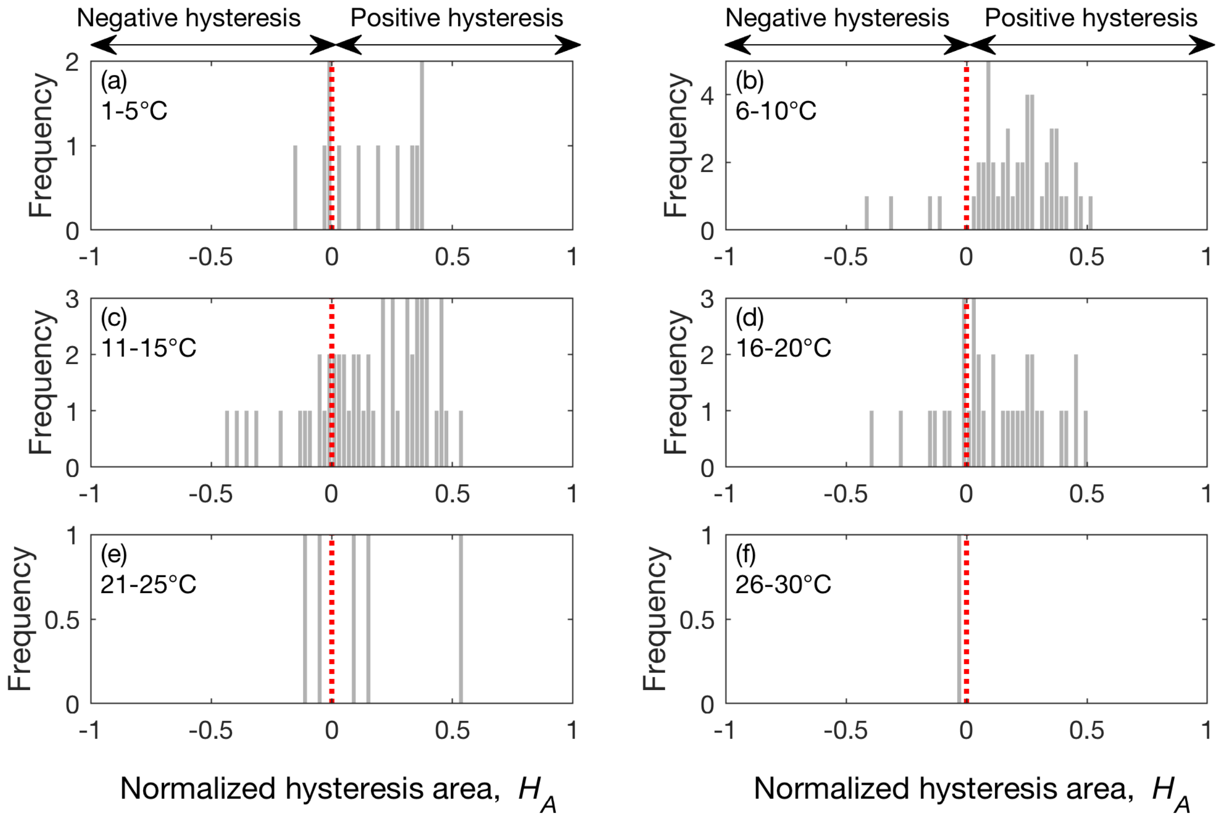

Supplemental Figure 8. **Predominantly positive seasonal CH_4 emission hysteresis (H_A) to air temperature is found across different range of mean seasonal air temperatures.** The distribution of normalized area of seasonal CH_4 emission hysteresis (H_A) to air temperature among site-years at individual mean seasonal temperature windows derived from the FLUXNET-CH_4 database. Each subpanel is evaluated for a different range of air temperatures. Positive seasonal CH_4 emission hysteresis indicates higher CH_4 emissions later in the frost-free season at the same temperature (e.g., Fig. 1d to 1f). Red dashed lines represent the y-axis in each data group (i.e., no hysteresis).

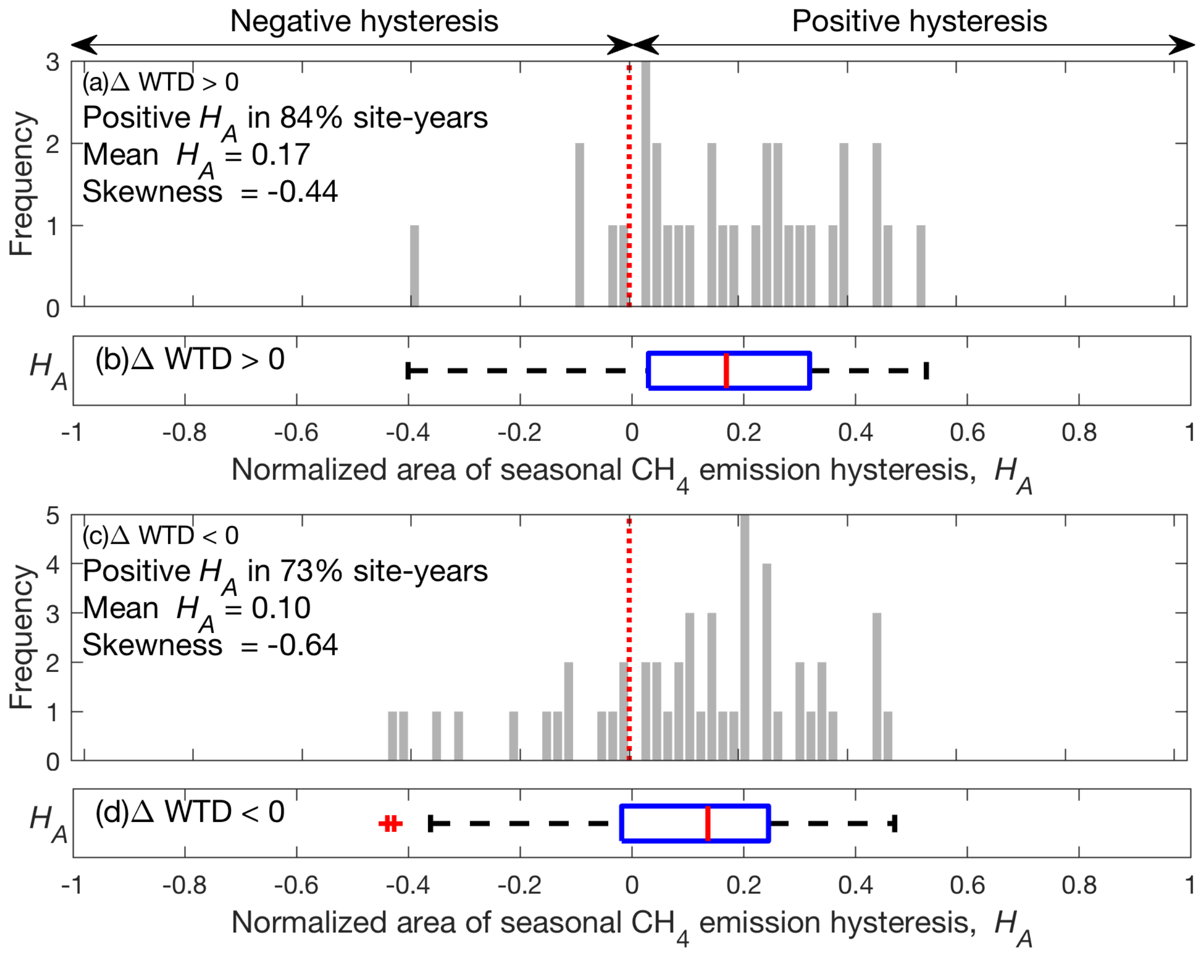

Supplemental Figure 9. **Predominantly positive seasonal CH_4 emission hysteresis (H_A) to air temperature is found across different wetness condition.** The distribution of normalized area of seasonal CH_4 emission hysteresis (H_A) to air temperature among site-years derived from the FLUXNET-CH_4 database when mean water table depth is higher (a) and lower (c) later in the frost-free season. Positive seasonal CH_4 emission hysteresis indicates higher CH_4 emissions later in the frost-free season at the same temperature (e.g., Fig. 1d to 1f). Red dashed lines represent the y-axis in each data group (i.e., no hysteresis). The corresponding boxplot of site-year specific H_A derived from the FLUXNET-CH_4 database (b, d). The red central mark, and the bottom and top edges of the blue box indicate the median, and the 25th and 75th percentiles, respectively. The black whiskers extend to the most extreme data points not considered outliers denoted in red plus symbol(s).

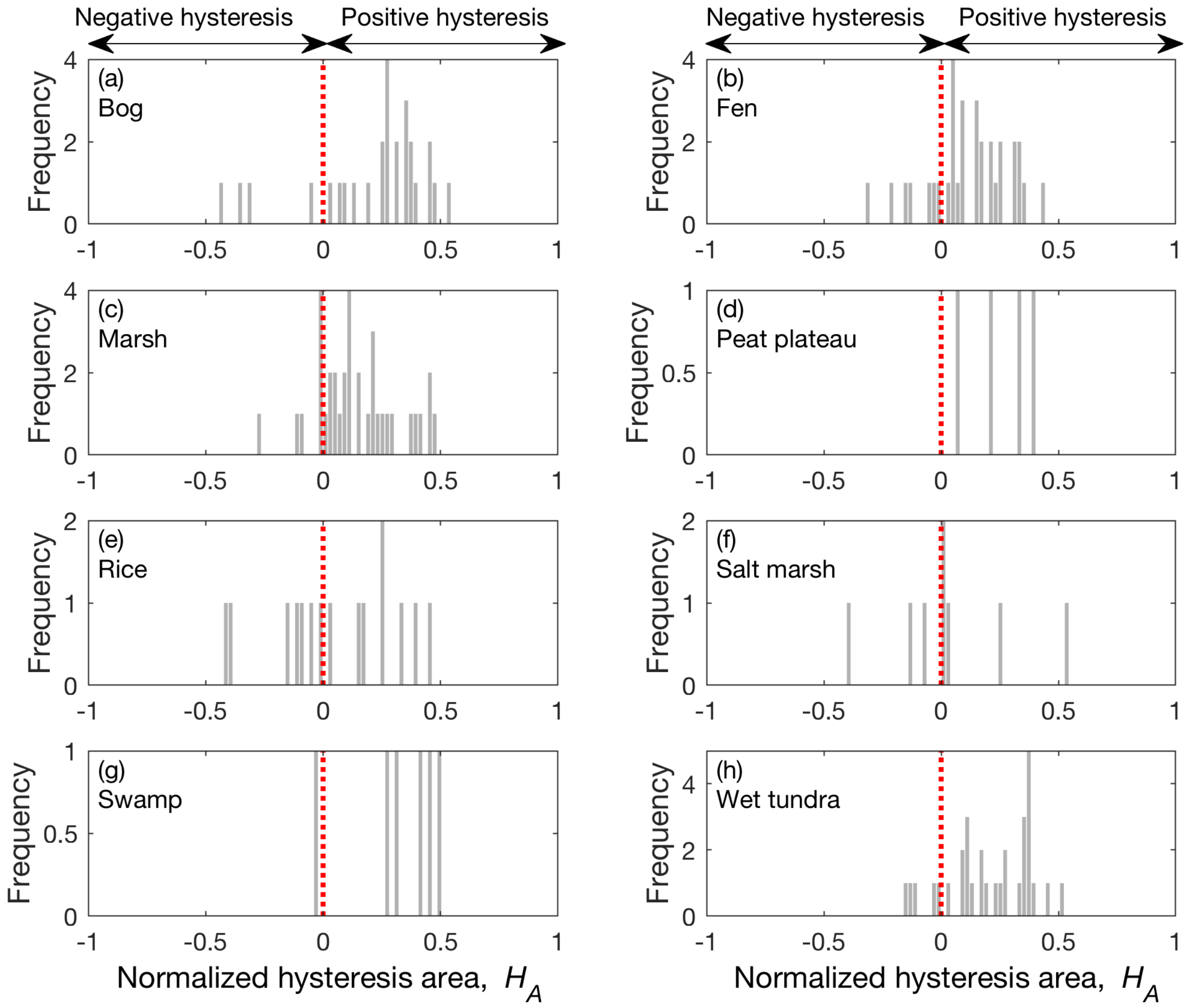

Supplemental Figure 10. **Predominantly positive seasonal CH_4 emission hysteresis (H_A) to air temperature is found across different ecosystem types.** The distribution of normalized area of seasonal CH_4 emission hysteresis (H_A) to air temperature among site-years at individual ecosystem types derived from the FLUXNET-CH_4 database. Positive seasonal CH_4 emission hysteresis indicates higher CH_4 emissions later in the frost-free season at the same temperature (e.g., Fig. 1d to 1f). Red dashed lines represent the y-axis in each data group (i.e., no hysteresis).

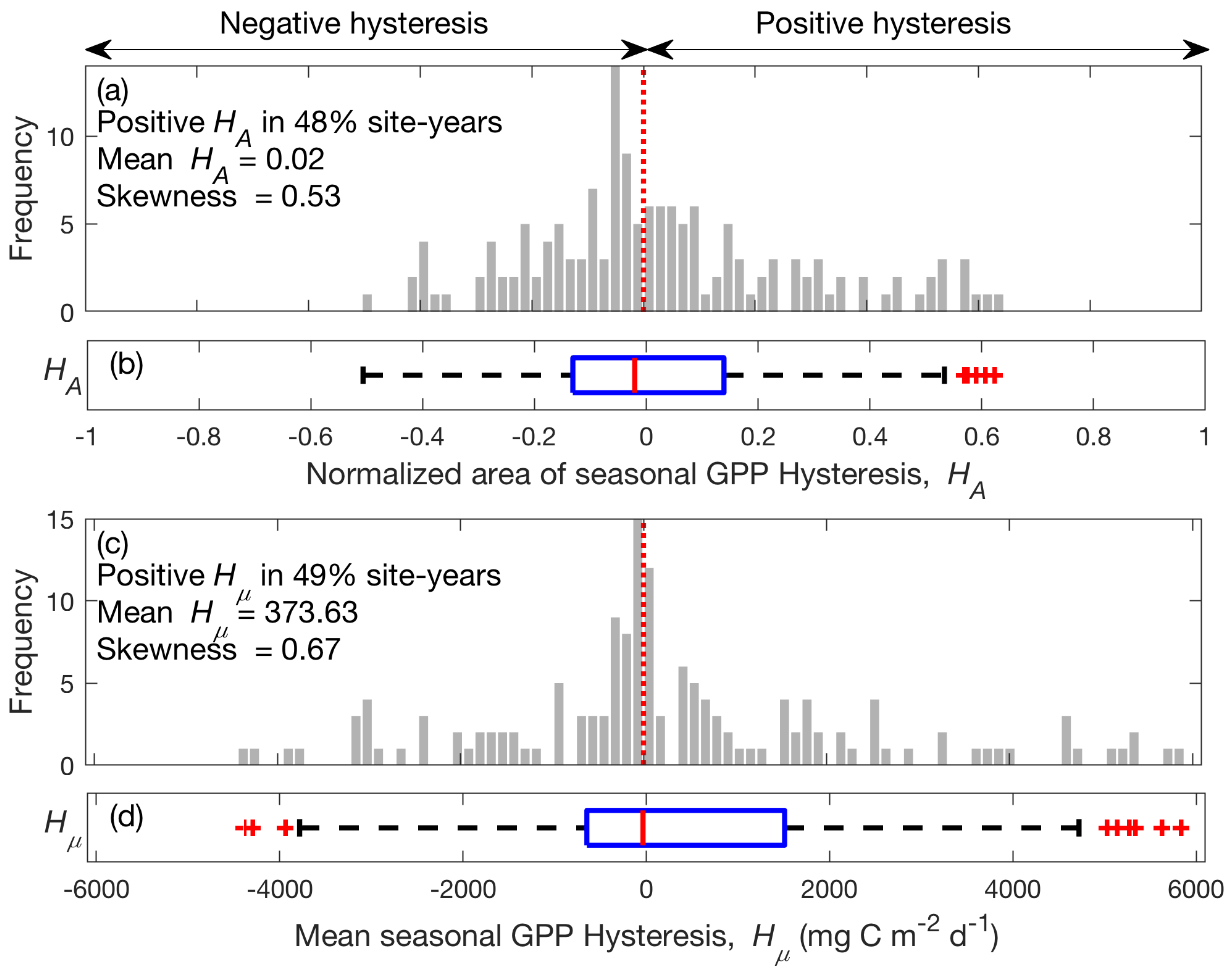

Supplemental Figure 11. **Intra-seasonal changes in emergent air temperature dependencies on gross primary productivity (GPP) show about equal site-year proportions of positive and negative seasonal GPP.** The distribution of mean seasonal gross primary productivity (GPP) hysteresis (a) and normalized area of seasonal GPP hysteresis (c) to air temperature among site-years derived from the FLUXNET-CH_4 database. Positive seasonal GPP hysteresis indicates higher GPP later in the frost-free season at the same air temperature. Red dashed lines represent the y-axis in each data group (i.e., no hysteresis). The corresponding boxplot of site-year specific mean seasonal GPP hysteresis (b) and normalized area of seasonal GPP hysteresis (d) derived from the FLUXNET-CH_4 database. The red central mark, and the bottom and top edges of the blue box indicate the median, and the 25th and 75th percentiles, respectively. The black whiskers extend to the most extreme data points not considered outliers denoted in red plus symbol(s).

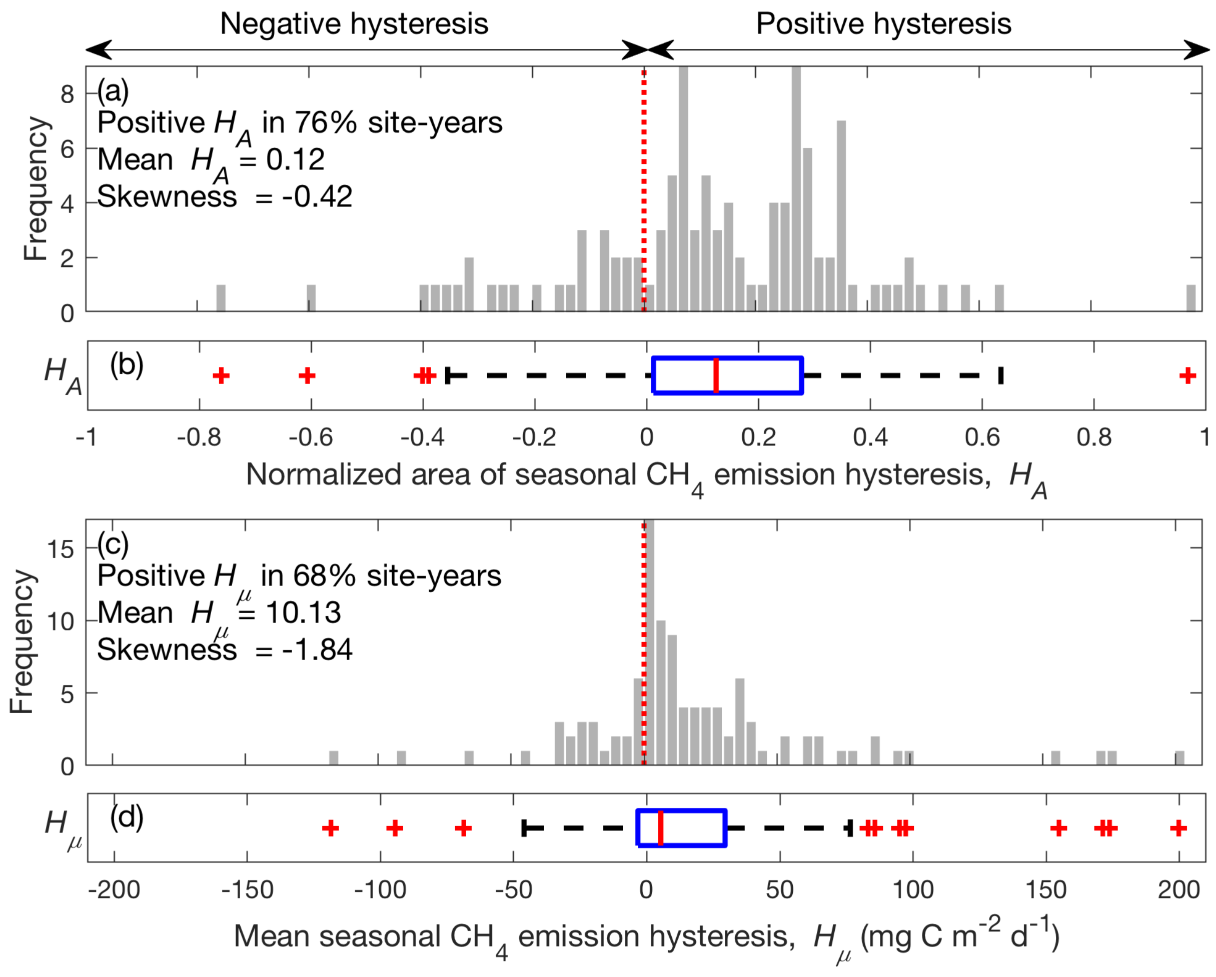

Supplemental Figure 12. **Predominantly positive seasonal CH_4 emission hysteresis to soil temperature measured at the shallowest soil layer among the FLUXNET-CH_4 site-years.** The distribution of mean seasonal CH_4 emission hysteresis (a) and normalized area of seasonal CH_4 emission hysteresis (c) to soil temperature measured at the shallowest soil layer among site-years derived from the FLUXNET-CH_4 database. Positive seasonal CH_4 emission hysteresis indicates higher CH_4 emission later in the frost-free season at the same soil temperature (e.g., Supplemental Figure 3). Red dashed lines represent the y-axis in each data group (i.e., no hysteresis). The corresponding boxplot of site-year specific mean seasonal CH_4 emission hysteresis (b) and normalized area of seasonal CH_4 emission hysteresis (d) derived from the FLUXNET-CH_4 database. The red central mark, and the bottom and top edges of the blue box indicate the median, and the 25th and 75th percentiles, respectively. The black whiskers extend to the most extreme data points not considered outliers denoted in red plus symbol(s).

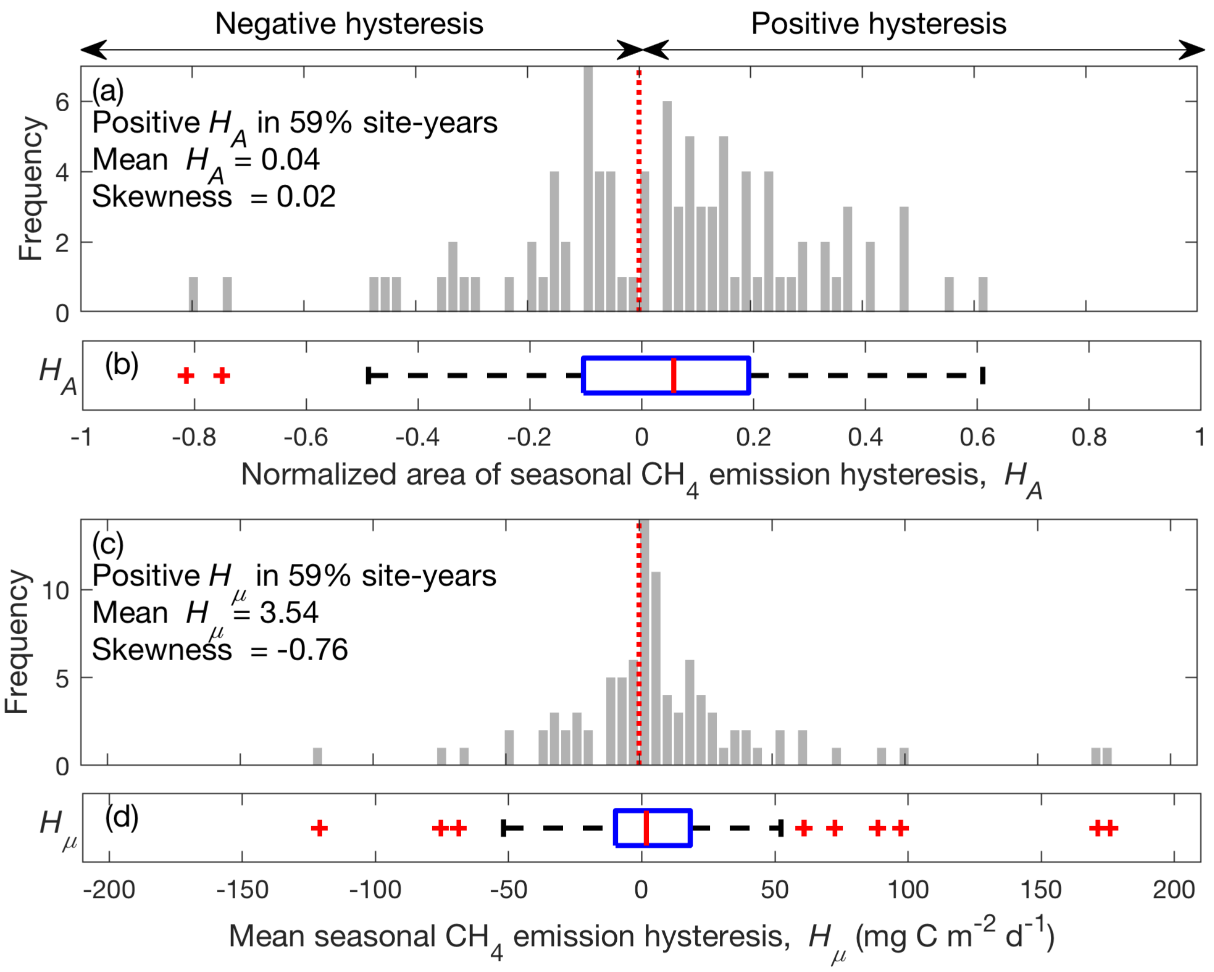

Supplemental Figure 13. **Predominantly positive seasonal CH_4 emission hysteresis to soil temperature measured at the deepest soil layer among the FLUXNET-CH_4 site-years.** The distribution of mean seasonal CH_4 emission hysteresis (a) and normalized area of seasonal CH_4 emission hysteresis (c) to soil temperature measured at the deepest soil layer among site-years derived from the FLUXNET-CH_4 database. Positive seasonal CH_4 emission hysteresis indicates higher CH_4 emission later in the frost-free season at the same soil temperature (e.g., Supplemental Figure 3). Red dashed lines represent the y-axis in each data group (i.e., no hysteresis). The corresponding boxplot of site-year specific mean seasonal CH_4 emission hysteresis (b) and normalized area of seasonal CH_4 emission hysteresis (d) derived from the FLUXNET-CH_4 database. The red central mark, and the bottom and top edges of the blue box indicate the median, and the 25th and 75th percentiles, respectively. The black whiskers extend to the most extreme data points not considered outliers denoted in red plus symbol(s).

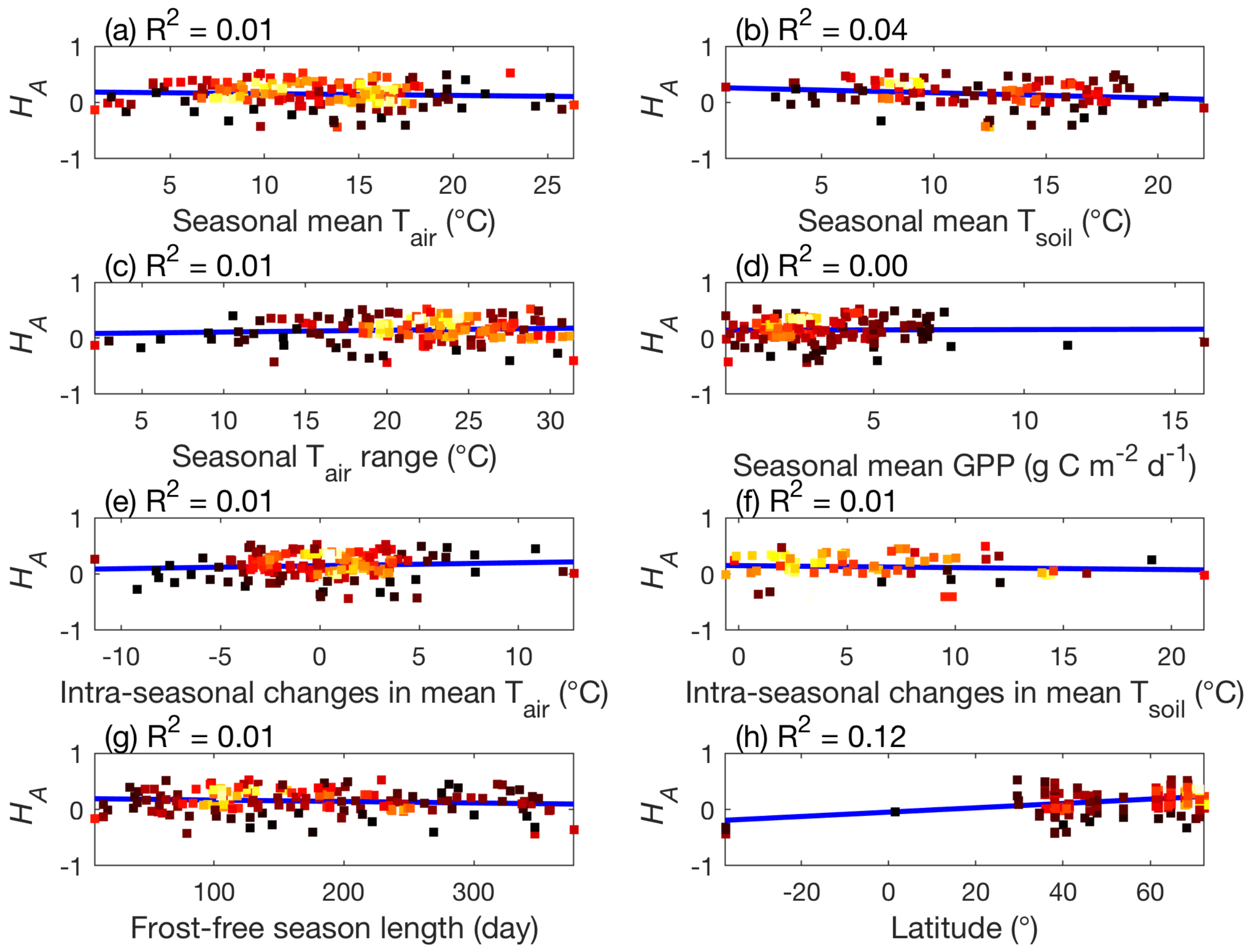

Supplemental Figure 14. **The observed seasonal CH_4 emission hysteresis is not sensitive to changes in temperature, gross primary productivity, frost-free season length and latitude.** The correlation between seasonal CH_4 emission hysteresis and seasonal mean air temperature (a), seasonal mean soil temperature (b), air temperature range measured in individual season (c), seasonal mean gross primary productivity (d), difference between mean air temperature measured in later and earlier parts of a frost-free season (e), difference between mean soil temperature measured in later and earlier parts of a frost-free season (f), forst-free season length (g), and latitude (h). Lighter colors in the density scatter plot represent denser data points. Solid blue lines represent the linear best fit.

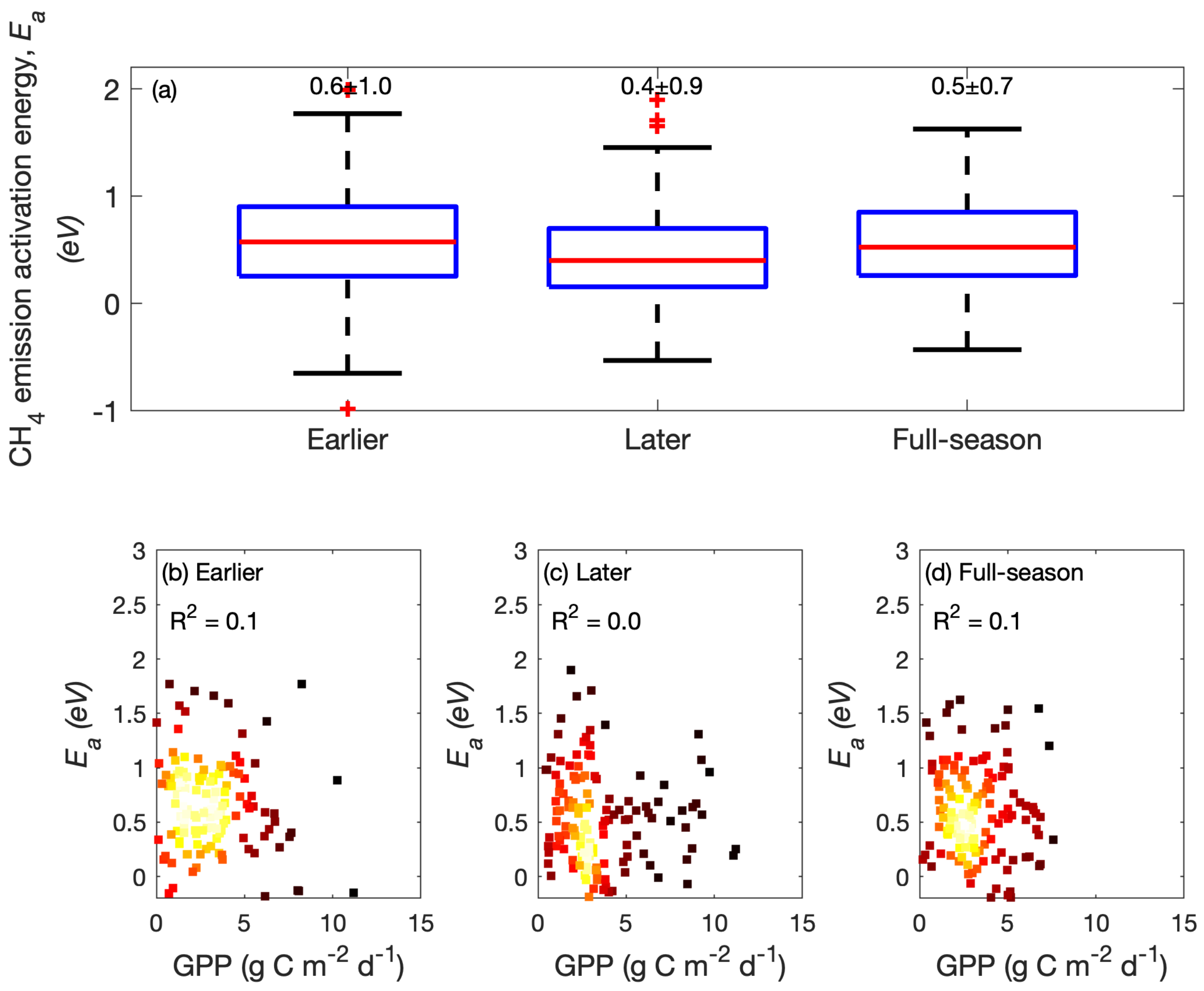

Supplemental Figure 15. **The apparent activation energy for CH_4 emission varies with the sampling period of the frost-free season.** The apparent activation energy for CH_4 emission inferred from the Boltzmann-Arrhenius equation (a), using measurements taken from the earlier, later, and full-season periods of a frost-free season across global wetland and rice paddy sites. The red central mark and open circle, and the bottom and top edges of the blue box indicate the median and mean, and the 25th and 75th percentiles, respectively. The black whiskers extend to the most extreme data points not considered outliers denoted in red plus symbol(s). Numbers above each boxplot indicate the mean ± standard deviation. Density scatter plots between gross primary productivity (GPP) and apparent activation energy for CH_4 emission (E_a) during the earlier (b), later (c), and full-season (d) periods of a frost-free season. Lighter colors in the density scatter plot represent denser data points.

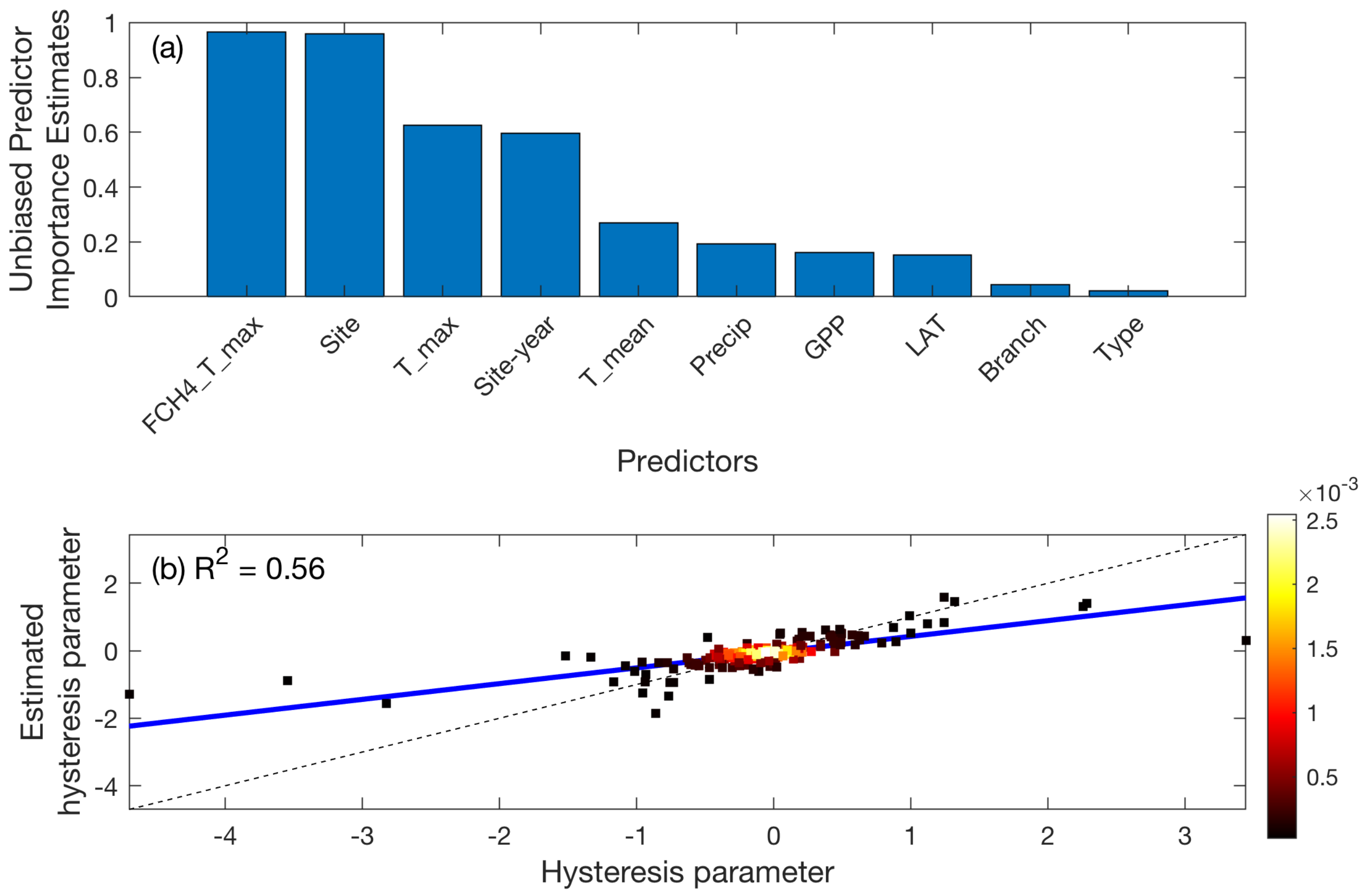

Supplemental Figure 16. **The accuracy of CH_4 emission predictions is strongly controlled by ecosystem-site variability.** The variable importance estimated by the random-forest model (a) and the performance of the estimated hysteresis parameter that quantifies the functional relationship between CH_4 emission and air temperature (b). Ten predictors were compared for their variable importance: earlier or later part in the frost-free season (Branch), gross primary productivity cumulated in each seasonal branch (GPP), precipitation cumulated in each seasonal branch (Precip), maximum seasonal temperature (T_max), mean temperature in each seasonal branch (T_mean), ecosystem type (Type), latitude (LAT), site (Site), site-year (Site-year) and CH_4 emission measured at maximum seasonal temperature (FCH4_T_max). Lighter colors in the density scatter plot (b) represent denser data points. Solid blue and dashed black lines represent the linear best fit and one to one lines, respectively.

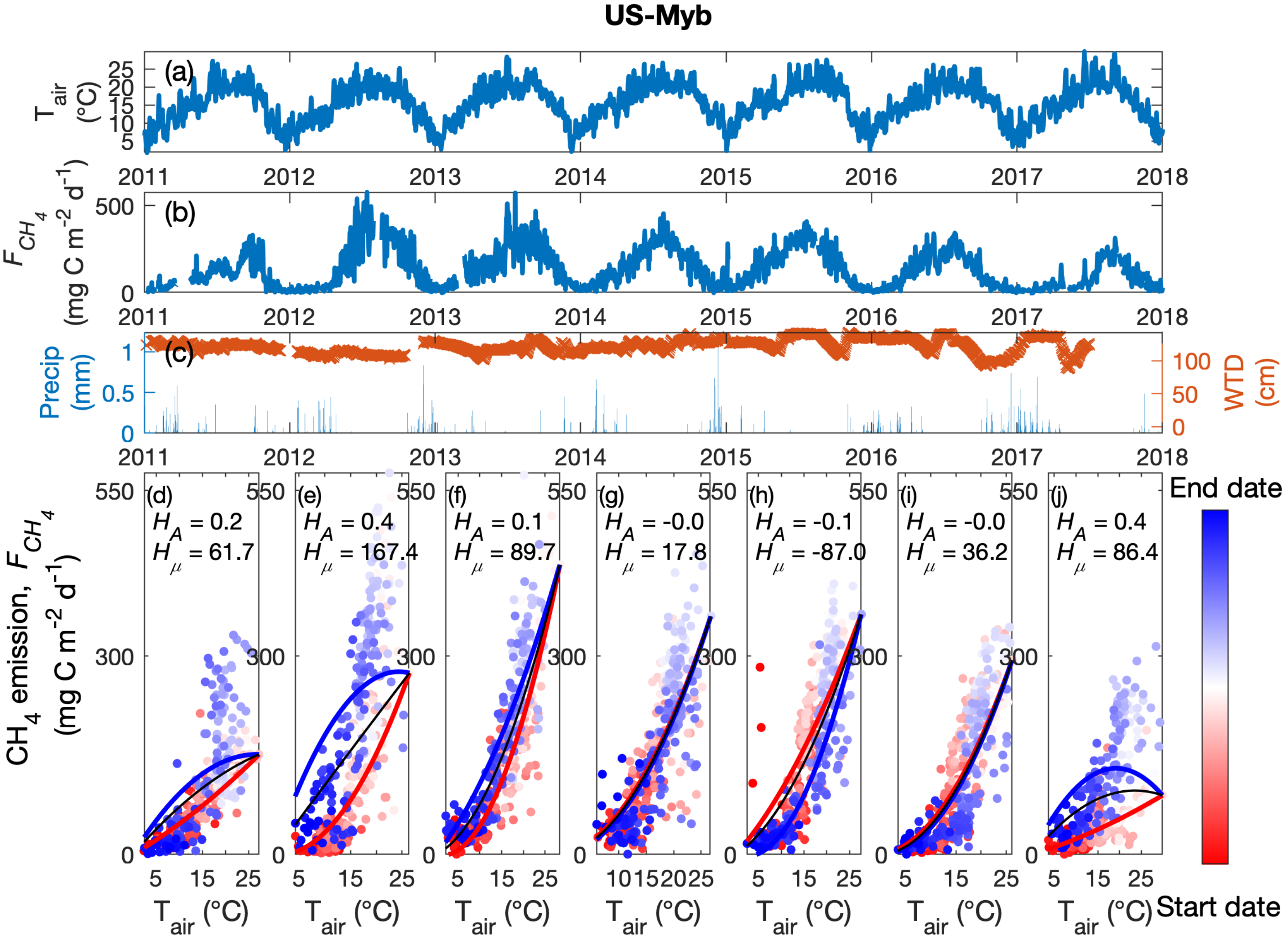

Supplemental Figure 17. **Seasonal CH_4 emission hysteresis shifts from positive to negative with increased salinity during 2014-2016.** The quality-controlled daily air temperature (a), CH_4 emissions (b), precipitation (c, left axis), and water table depth (c, right axis) measured at the Sacramento-San Joaquin Delta of California in USA (US-Myb) from 2011 to 2017. CH_4 emission air temperature dependencies (lines) derived from daily measurements (dots) recorded at US-Myb from 2011 (d) to 2017 (j). The results inferred from earlier and later parts of the frost-free season, and full frost-free season are colored in red, blue, and black, respectively. Start and end dates represent the beginning and ending of the frost-free season, respectively. The seasonal CH_4 emission hysteresis rotates from positive (2011-2013), neutral (2014 and 2016), to negative (2015), which may be related to the increased salinity during 2014-2016.

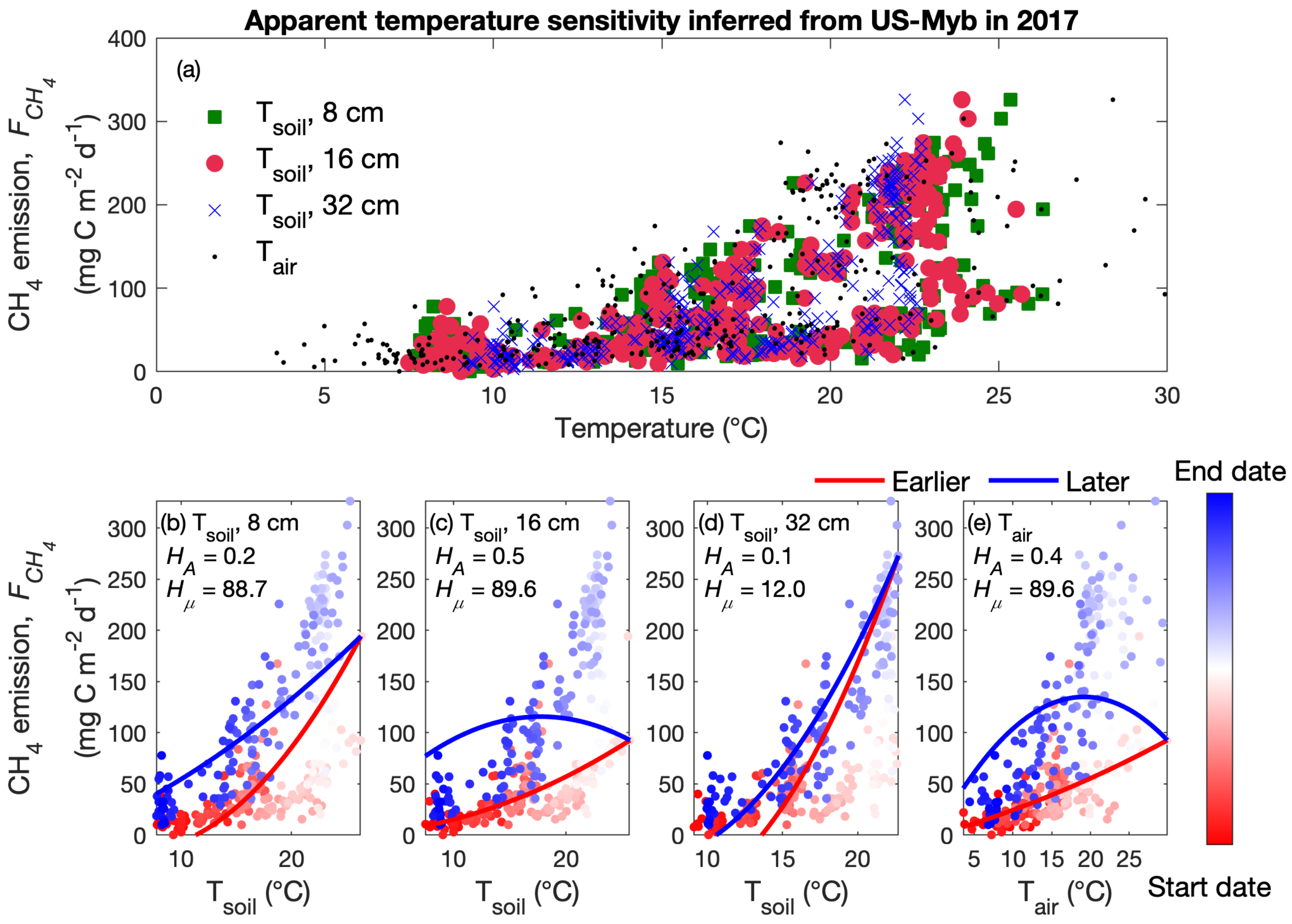

Supplemental Figure 18. **The magnitude of seasonal CH_4 emission hysteresis varies non-monotonically along the soil profile.** The emergent dependence of CH_4 emission on air and soil temperatures inferred from measurements taken at the Sacramento-San Joaquin Delta of California in USA (US-Myb) in 2017 (a). Daily measurements of CH_4 emission and temperature for soil temperature measured at 8 cm (b), for soil temperature measured at 16 cm (c), for soil temperature measured at 32 cm (d), and for air temperature (e). The results inferred from earlier and later parts of the frost-free season are colored in red and blue, respectively. Start and end dates represent the beginning and ending of the frost-free season, respectively.

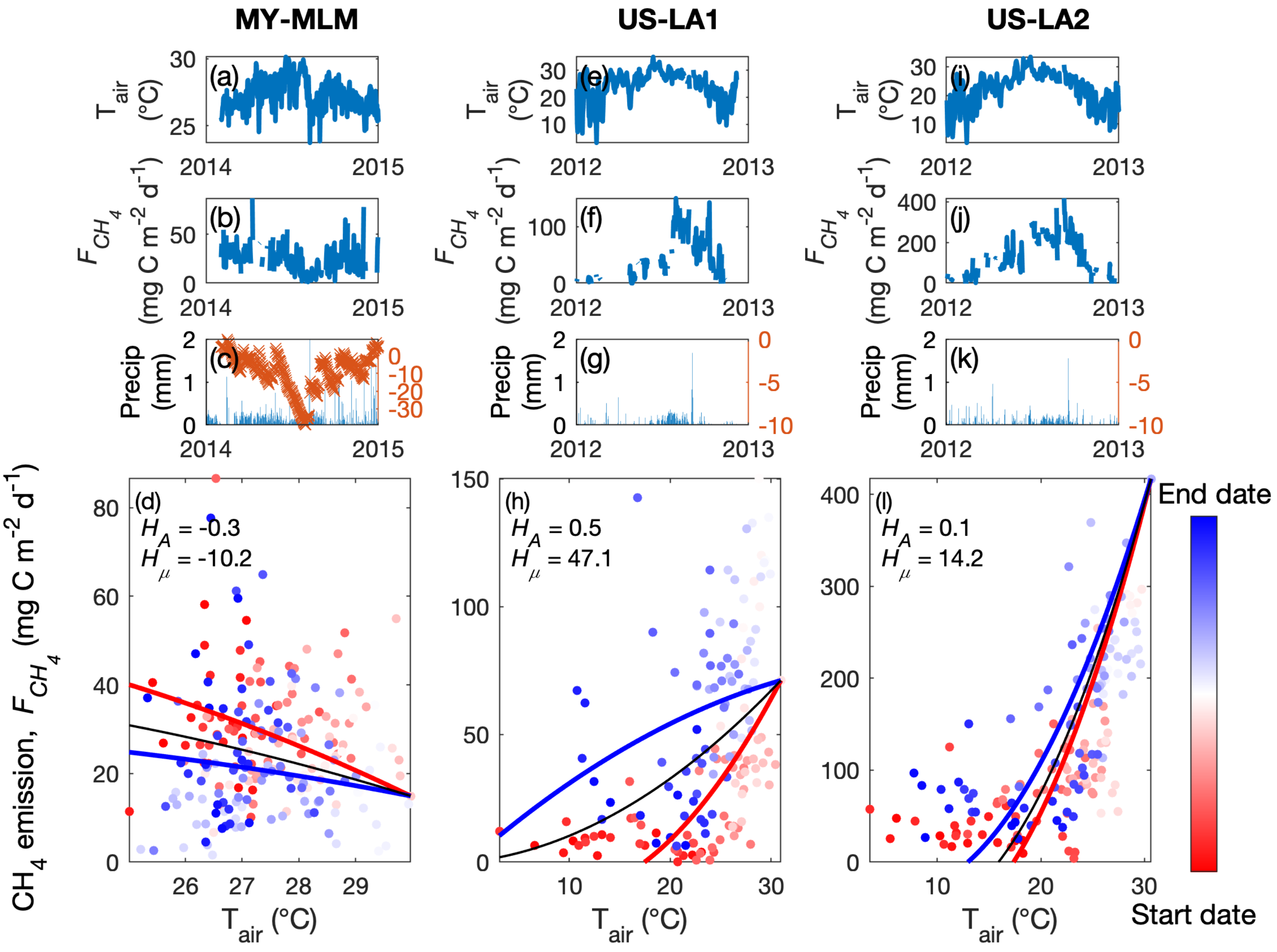

Supplemental Figure 19. **Intra-seasonally varying emergent air temperature dependence on CH_4 emission is detected in tropical and subtropical sites.** The quality-controlled daily air temperature (a, e, i) and CH_4 emissions (b, f, j), precipitation (c, g, k, left axis), and water table depth (c, g, k, right axis) measured at Maludam swamp in Malaysia (MY-MLM), Brackish marsh in the US (US-LA1), and Freshwater marsh in the US (US-LA2), respectively. CH_4 emission air temperature dependencies (lines) derived from daily measurements (dots) recorded at MY-MLM for 2014 (d), at US-LA1 for 2012 (h), and at US-LA2 for 2012 (l). The results inferred from earlier and later parts of the frost-free season, and full frost-free season are colored in red, blue, and black, respectively. Start and end dates represent the beginning and ending of the frost-free season, respectively. H_μ and H_A denote the mean seasonal CH_4 emission hysteresis and normalized area of seasonal CH_4 emission hysteresis calculated in each site-year, respectively.

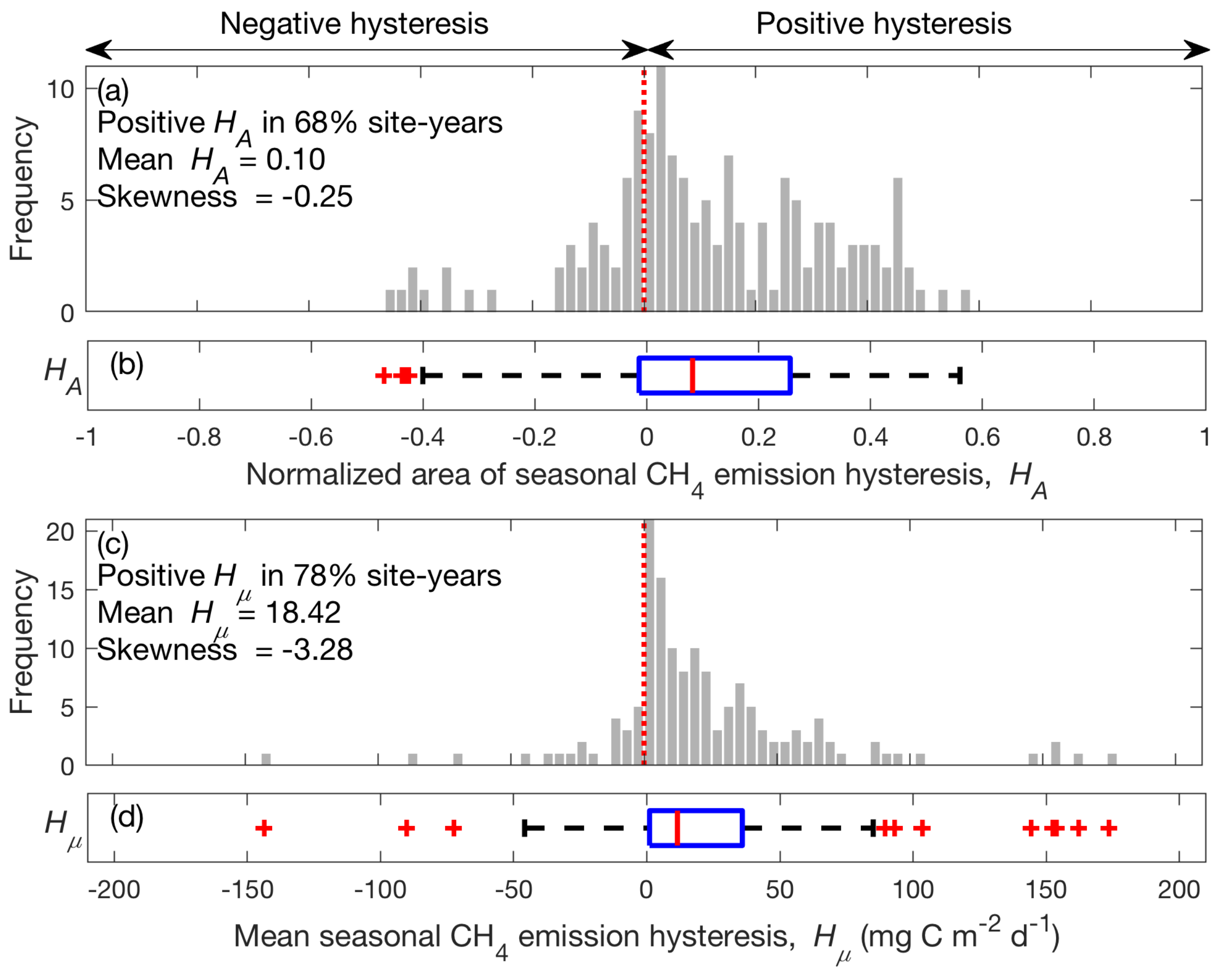

Supplemental Figure 20. **The distribution of seasonal CH_4 emission hysteresis inferred from the period when gross primary productivity (GPP) is above zero is consistent with the patterns found using data collected during the frost-free season.** The distribution of mean seasonal CH_4 emission hysteresis (a) and normalized area of seasonal CH_4 emission hysteresis (c) to air temperature among site-years derived from the FLUXNET-CH_4 database, when GPP > 0. Positive seasonal CH_4 emission hysteresis indicates higher CH_4 emission later in the frost-free season at the same air temperature (e.g., Figure 1). Red dashed lines represent the y-axis in each data group (i.e., no hysteresis). The corresponding boxplot of site-year specific mean seasonal CH_4 emission hysteresis (b) and normalized area of seasonal CH_4 emission hysteresis (d) derived from the FLUXNET-CH_4 database. The red central mark, and the bottom and top edges of the blue box indicate the median, and the 25th and 75th percentiles, respectively. The black whiskers extend to the most extreme data points not considered outliers denoted in red plus symbol(s).

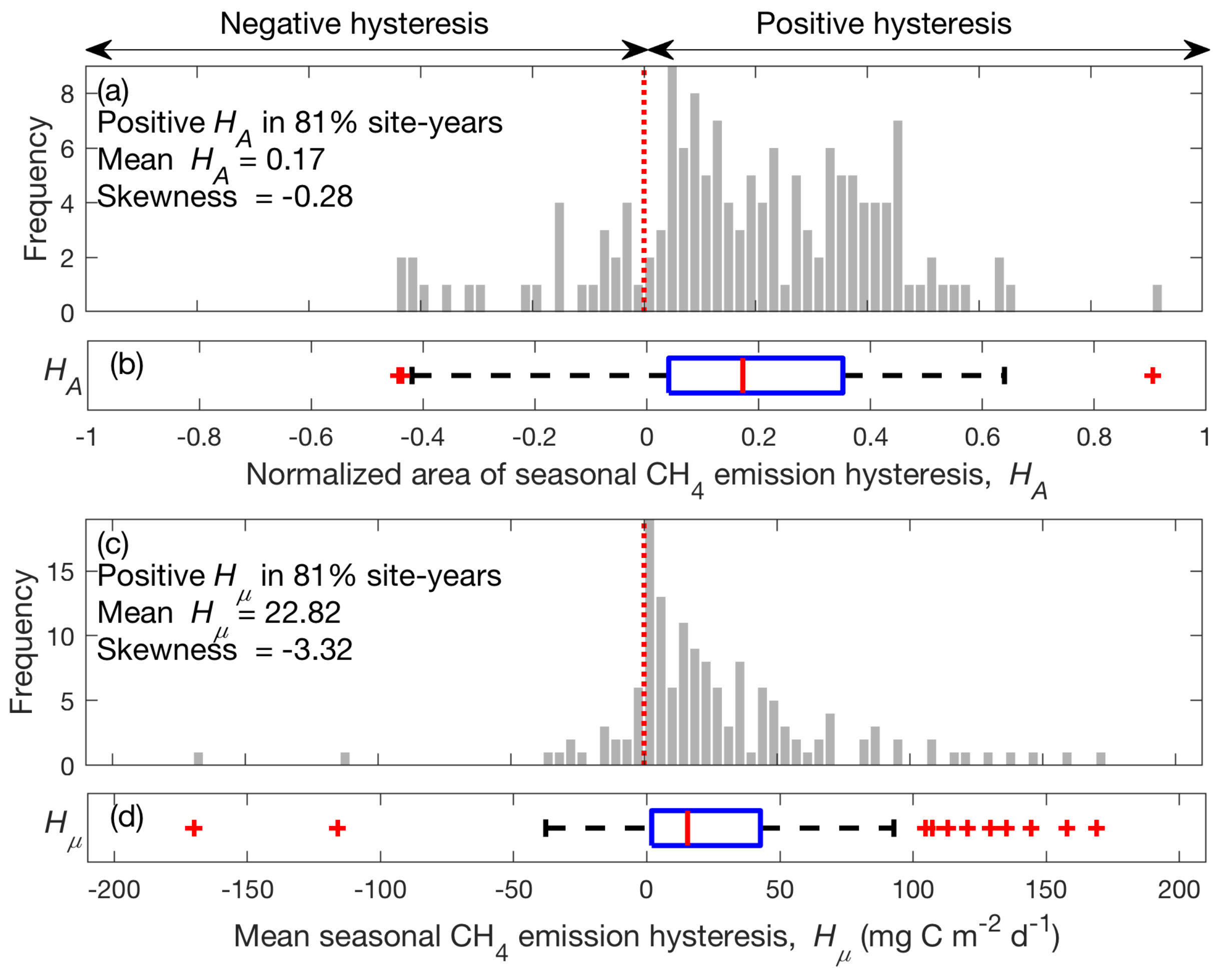

Supplemental Figure 21. **The distribution of seasonal CH_4 emission hysteresis inferred from the period when gross primary productivity (GPP) is above 5% of annual GPP maximum is consistent with the patterns found using data collected during the frost-free season.** The distribution of mean seasonal CH_4 emission hysteresis (a) and normalized area of seasonal CH_4 emission hysteresis (c) to air temperature among site-years derived from the FLUXNET-CH_4 database, when GPP > 5% of annual GPP maximum. Positive seasonal CH_4 emission hysteresis indicates higher CH_4 emission later in the frost-free season at the same air temperature (e.g., Figure 1). Red dashed lines represent the y-axis in each data group (i.e., no hysteresis). The corresponding boxplot of site-year specific mean seasonal CH_4 emission hysteresis (b) and normalized area of seasonal CH_4 emission hysteresis (d) derived from the FLUXNET-CH_4 database. The red central mark, and the bottom and top edges of the blue box indicate the median, and the 25th and 75th percentiles, respectively. The black whiskers extend to the most extreme data points not considered outliers denoted in red plus symbol(s).

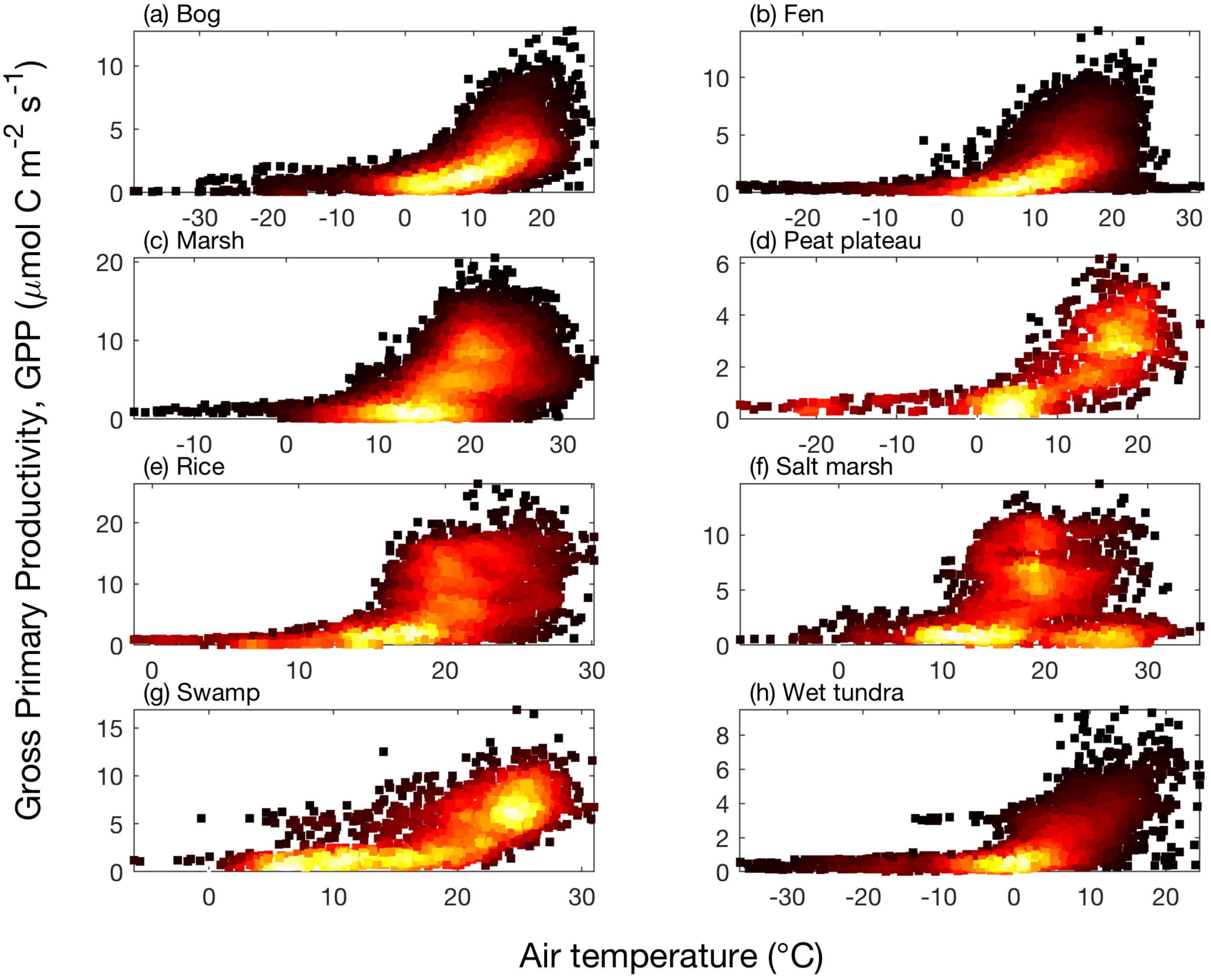

Supplemental Figure 22. **Substantial GPP values are detected when air temperatures are well below 0 ˚C.** Density scatter plots between gross primary productivity (GPP) and air temperature measured at the bog (a), fen (b), marsh (c), peat plateau (d), rice paddy (e), salt marsh (f), swamp (g), and wet tundra (h) sites, when gross primary production (GPP) is greater than 5% of annual GPP maximum.

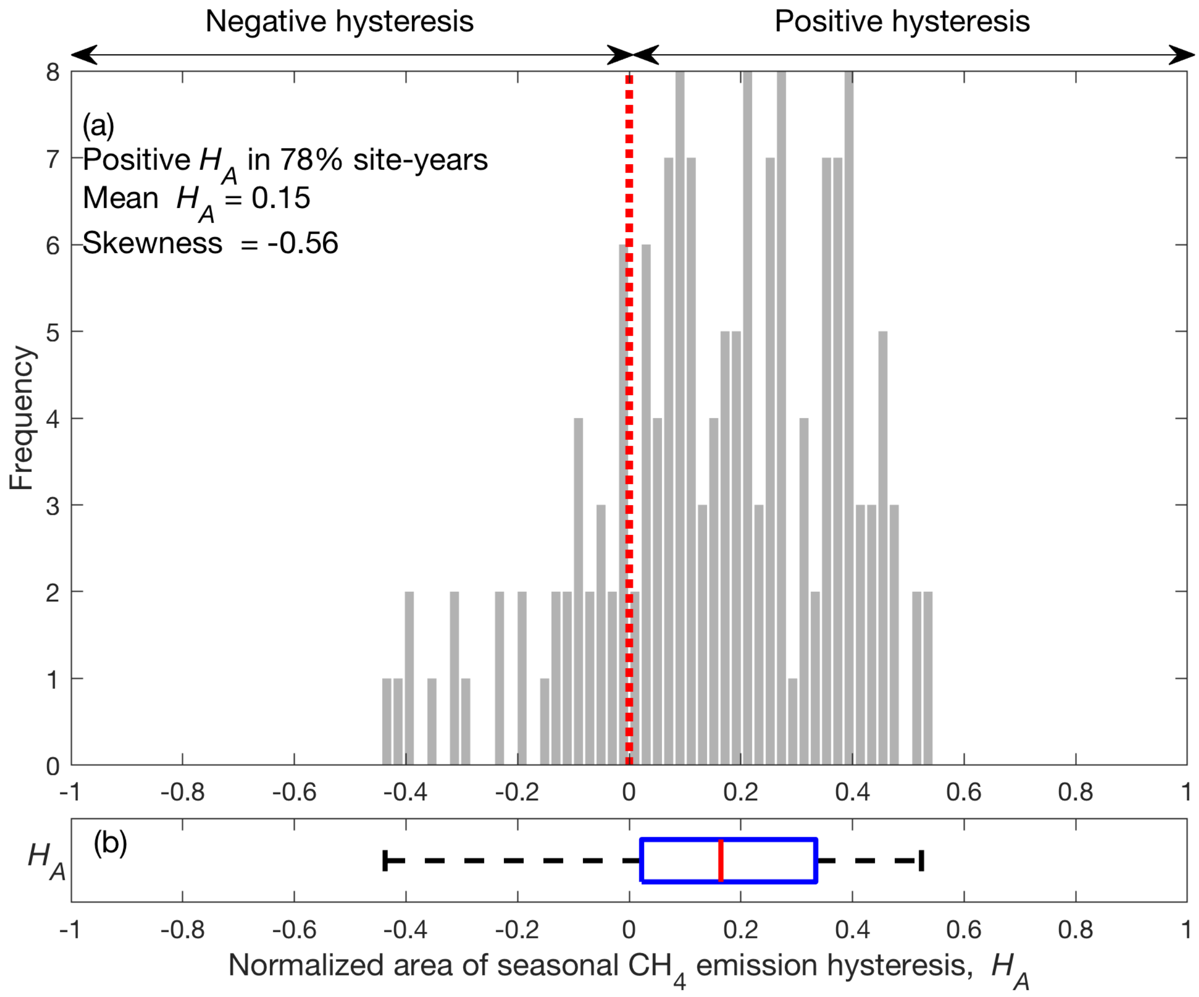

Supplemental Figure 23. **The distribution of seasonal CH_4 emission hysteresis inferred from non-zero CH_4 emissions at 0 °C is consistent with the patterns found assuming no CH_4 emissions at 0 °C.** The distribution of normalized area of seasonal CH_4 emission hysteresis (H_A) to air temperature among site-years derived from the FLUXNET-CH_4 database, when mean CH_4 emission between -0.5 to 0.5 °C is used to represent CH_4 emissions at 0 °C (a). Positive seasonal CH_4 emission hysteresis indicates higher CH_4 emissions later in the frost-free season at the same temperature (e.g., Fig. 1d to 1f). Red dashed lines represent the y-axis in each data group (i.e., no hysteresis). The corresponding boxplot of site-year specific H_A derived from the FLUXNET-CH_4 database (b). The red central mark, and the bottom and top edges of the blue box indicate the median, and the 25th and 75th percentiles, respectively. The black whiskers extend to the most extreme data points not considered outliers denoted in red plus symbol(s).
